# Safety assessment of *Enterococcus lactis* strains complemented with comparative genomics analysis reveals probiotic and safety characteristics of the entire species

**DOI:** 10.1186/s12864-023-09749-9

**Published:** 2023-11-06

**Authors:** Noha A. Ahmed, Rania Abdelmonem Khattab, Yasser M. Ragab, Mariam Hassan

**Affiliations:** 1 Department of Microbiology and Immunology, Faculty of Pharmacy, Cairo University, Kasr El-Aini Street, Cairo, 11562 Egypt; 2 Department of Microbiology and Immunology, Faculty of Pharmacy, Galala University, New Galala City, Suez 43511 Egypt

**Keywords:** *Enterococcus faecium*, Gut microbiota; Human stool, Probiotics, Whole genome sequencing, CRISPR-cas

## Abstract

**Background:**

The gut microbiota is considered a rich source for potential novel probiotics. *Enterococcus* genus is a normal component of a healthy gut microbiota, suggesting its vital role. Nosocomial infections caused mainly by *E. facalis* and *E. faecium* have been attributed to the plasticity of the *Enterococcus* genomes. In this study, we assessed the probiotic and safety characteristics of two *E. lactis* strains isolated from the human gut microbiota using *in-vitro* and in silico approaches. Additionally, the safety of the *E. lactis* species was evaluated using comparative genomics analysis.

**Results:**

The two *E. lactis* strains 10NA and 50NA showed resistance to bile salts and acid tolerance with antibacterial activity against *Escherichia coli*, *Salmonella typhi*, and *Clostridioides difficile.* For safety assays, the two strains did not display any type of hemolysis on blood agar, and the survival of Caco-2 cells was not significantly different (*P*-value > 0.05) compared to the control using cell free supernatants at 100% (v/v), 50% (v/v), 10% (v/v), and 5% (v/v) concentrations. Regarding antibiotic susceptibility, both strains were sensitive to vancomycin, tetracycline, and chloramphenicol. Comprehensive whole-genome analysis revealed no concerning associations between virulence or antibiotic resistance genes and any of the identified mobile genetic elements. Comparative genome analysis with closely related *E. faecium* species genomes revealed the distinctive genomic safety of the *E. lactis* species.

**Conclusions:**

Our two *E. lactis* strains showed promising probiotic properties *in-vitro*. Their genomes were devoid of any transferable antibiotic resistance genes. In silico comparative analysis confirmed the safety of the *E. lactis* species. These results suggest that *E. lactis* species could be a potential source for safer *Enterococcus* probiotic supplements.

**Supplementary Information:**

The online version contains supplementary material available at 10.1186/s12864-023-09749-9.

## Introduction

The word “probiotics” is derived from the Latin word “pro” (for) and the Greek word “bios” (life), meaning for life. The history of beneficial microorganisms goes back to the use of fermented food. However, the first link between probiotic consumption and enhanced longevity was made by Elie Metchnikoff at the end of the 19^th^ century [1]. Probiotics were defined as “live microorganisms that, when administered in adequate amounts, confer a health benefit on the host” [2].

The selection of new probiotic strains starts with isolation from different ecological commensal microbial communities. It is preferable that probiotics for human use to be isolated from human or food products to ensure their safety and ability to colonize human intestinal mucosa. Accordingly, human stool, breast milk, fermented products, and animal-origin food are reliable sources for the isolation of potential probiotic strains [3]. The customary method for the selection of potential probiotics starts with a series of *in-vitro* tests that include tolerance to acid stress, resistance to bile salts, adherence to epithelial cells, and antagonistic effects against certain pathogens [4, 5].

The most important step in the selection of a potential probiotic strain is to fully assess its safety profile. Although there is no generally accepted approach recommended, it is agreed that safety assessment begins with the correct identification of the potential probiotic strain. Identification could be done using both phenotypic and genotypic methods. While phenotypic methods may be used for initial screening, genotypic methods are mandatory. Using whole-genome sequencing is a fast method for screening for antibiotic resistance and virulence expressing genes. The whole-genome analysis is considered a tool to predict non-expressed risk factors [6].

*Lactobacillus* and *Bifidobacterium* species are the most common probiotics in the market. These two genera are generally recognized as safe by the FDA and EFSA [7]. On the contrary, the *Enterococcus* genus has a notorious reputation due to the rise in nosocomial infections [8]. *Enterococcus* species are important components of a healthy microbiota and should not be excluded completely from probiotic supplements due to the pathogenicity of a few species [9–11]. The main cause of *Enterococcus* pathogenicity is the plasticity of their genomes and their ability to accept mobile genetic elements [12]. Prokaryotic genomes exhibit both innate and adaptive immune systems, represented by restriction endonucleases and CRISPR-Cas systems, respectively. However, the interplay between these two systems requires further investigation [13]. The ability of bacteria to accept foreign mobile elements is a critical evolutionary concern, as they must distinguish between harmful lytic viruses and conjugative mobile elements that can transfer advantageous traits for bacterial fitness [14]. The adaptability and genomic plasticity of *Enterococcus* species confer them with evolutionary advantages. Nonetheless, they also harbor the potential to acquire and transfer antibiotic resistance genes (ARGs), representing a darker aspect of their genome plasticity [15, 16].

Probiotic traits and safety profiles are strain-specific. Consequently, the discovery of new strains may reveal better properties or novel effects than existing ones. In this study, two *E. lactis* strains named 10NA and 50NA were isolated from the human gut microbiota. Assessments of the probiotic properties and safety profiles were performed using phenotypic and genotypic methods. Also, the safety of *E. lactis* species was thoroughly investigated using comparative genomics analysis.

## Materials and methods

### Isolation of potential probiotic candidates

Isolated bacterial communities from fecal samples collected in a previous study by RA Khattab, NA Ahmed, YM Ragab and SA Rasmy [17] were used for screening for potential probiotics. Briefly, a total of 123 fecal samples were collected from different human subjects (Fig. S1). The bacterial communities maintained from these samples were cultured anaerobically on blood agar plates (Neogen Co., USA) supplemented with 0.05% cysteine-HCl (SERVA, Germany). Colonies with different morphologies showing no hemolysis were subcultured on de Man, Rogosa, and Sharpe (MRS) agar (Neogen Co., USA) supplemented with 0.05% cysteine-HCl for purification and further evaluation. All isolated strains were maintained in Brain Heart Infusion (BHI) broth (Oxoid, UK) with 20% glycerol at -80 °C. The isolated strains were inspected under a microscope (Olympus, USA) for Gram staining and morphology [18]. The Gram staining was performed according to the method described by N Tripathi and A Sapra [19].

### Bacterial strains and growth conditions

The reference bacterial strains included in this study were: *Escherichia coli* O157:H7 EDL933, *Salmonella typhi* ATCC 35664, and *Clostridioides difficile* C74A clinical isolate. C. *difficile* was cultured in Reinforced Clostridial Medium (RCM) semi-broth (Oxoid, UK) at 37 °C for 24 h under anaerobic conditions in an anaerobic jar with Anaerogen gas packs. *E. coli* and *S. typhi* were cultured in Muller-Hinton (MH) broth (Oxoid, UK) at 37 °C for 24 h under aerobic conditions. All strains were maintained in BHI broth with 20% glycerol at -80 °C.

### Probiotic properties assessment

#### Assessment of tolerance to acidic environment and bile salts resistance

Acid and bile salts tolerance assays were conducted according to the method used by HM Elzeini, ARAA Ali, NF Nasr, M Hassan, AAm Hassan and YE Elenany [20], with minor modifications. In the acid resistance assay, 3 ml of MRS medium at pH 3 or the control (MRS medium at pH 6.5) were inoculated with 300 μl (10% v/v) overnight cultures pre-adjusted to an OD600 of 0.1. The inoculated media were then incubated at 37 °C under microaerophilic conditions using a 5% carbon dioxide incubator (BINDER, Germany) [21]. Samples (30 μl) were taken at zero, 1.5 h, and 3 h.The bile resistance assay was conducted similarly to the acid resistance assay. In this case, 3 ml of MRS medium supplemented with either 0.3% w/v or 0.7% w/v of a bile salts mixture (Loba Chem, India), or the control (MRS medium), were inoculated with 300 μl (10% v/v) overnight cultures pre-adjusted to an OD600 of 0.1. The inoculated media were then incubated under the same conditions. Samples (30 μl) were taken at zero, 1.5 h, 3 h, 6 h, and 24 h.

Serial dilutions of 10-fold were performed for the samples in sterile peptone saline. Subsequently, 10 μl of each dilution was spotted on MRS agar plates and incubated at 37 °C for 48 h in a 5% carbon dioxide incubator. Growth was monitored using the plate count method, and viable counts were expressed as CFU/ml. Acid tolerance was determined by comparing the plate count after 1.5 h and 3 h with the initial plate count at zero time. The results were expressed as an average percentage of survival. Bile tolerance was determined by comparing the growth curves at 0.3% (w/v) and 0.7% (w/v) bile salts concentrations with the growth curve of the control (0% w/v). The two assays were performed in triplicate and recorded as the mean ± standard deviation (SD).

#### Antagonistic activity of isolated strains against pathogenic bacteria

The antibacterial activity of the isolates was determined by the agar overlay method against *E. coli* O157:H7 EDL933, *S. typhi* ATCC 35664, and *C. difficile* clinical isolate. Each of the individual probiotic strains was spot inoculated onto MRS agar plates and incubated at 37 °C for 48 h in a 5% CO_2_ incubator. The MRS agar plates containing the growth of probiotic candidates ‘in spot form’ (≈6 mm diameter) were thereafter overlaid with MH agar (0.8% agar) containing a single indicator strain (*E. coli* or *S. typhi*) in the individual plates and incubated at 37 °C for 24 h under aerobic conditions [22]. Regarding *C. difficile*, the spotted MRS plates were overlaid with RCM ager (1.5% agar) containing the indicator strain and incubated at 37 °C for 24 h under anaerobic conditions in an anaerobic jar with Anaerogen gas packs at 37 °C for 24 h [17, 23].

### Phenotypic safety profiling

#### Antibiotic susceptibility

The antibiotic susceptibility test was performed following the disc diffusion method [24, 25], as described before by Halder and Mandal [22], using MRS agar and approximately10^8^ CFU inoculum from the probiotic strains. The antibiotic discs (Bioanalyse, Turkey) used were Gentamicin (CN: 10-µg/disc), Tetracycline (TE: 30-µg/disc), Chloramphenicol (C: 30-µg/disc), Clindamycin (DA: 2-μg/disc), Erythromycin (E: 15-µg/disc), Kanamycin (K: 30-µg/disc), Streptomycin (S: 10-µg/disc) and Vancomycin (VA: 30-µg/disc). The determined zone diameter of inhibition (ZDI) values were interpreted according to the cut-off points given by the CLSI document [26].

#### Cytotoxicity/anti-proliferative activity assay using human colon Caco-2 cell line

The human colonic tumor-derived epithelial cell line Caco-2 was purchased from the Egyptian Holding Company for Biological Products and Vaccines (VACSERA, Egypt). The Caco-2 cell line was cultured in Dulbecco’s Modified Eagle Medium (Gibco, USA) supplemented with 10% fetal bovine serum, 100 µg/mL penicillin, and 100 µg/ml streptomycin. The cells were maintained in tissue culture flasks (Griener, Germany) in a humidified 5% CO2 incubator at 37 °C until confluent. The detachment of cells was done using 0.25% Trypsin–EDTA (AMRESCO, USA). The cell free supernatants (CFSs) for the two strains were prepared by centrifuging the overnight cultures in MRS broth at 6000 rpm for 15 min. Then the supernatants were filtered sterilized using a 0.22 μm cellulose acetate syringe filter and kept at -20 °C until use. The cytotoxic effect of the produced metabolites by the isolated strains on the human colon adenocarcinoma cell line (Caco-2) was determined using MTT assay [27, 28]. Briefly, Caco-2 cell line monolayers were seeded into 96-well plates (1 × 10^4^ cells / well) with complete culture media and incubated overnight. Then the culture media was aspirated, and the Caco-2 cells were subjected to the CFSs at 100% (v/v), 50% (v/v), 10% (v/v), and 5% (v/v) concentrations. In addition, sterile MRS broth was added to the Caco-2 cell at the same concentrations to serve as the control. The dilutions were performed in triplicates. The test and control were incubated at a 5% CO2 incubator at 37 °C for 48 h. After that, dead cells were washed out using phosphate buffer saline, pH 7.2 ± 0.2 (PBS-0.05% Tween). Residual live cells were treated with 0.5% MTT stain as 25 µl/ well. Plates were incubated for 3–4 h at 37 °C. Developed intra-cytoplasmic MTT formazan crystals were dissolved in 0.05 ml of dimethyl sulfoxide (DMSO) for 30 min on a plate shaker. Optical densities at 570 nm were read using (Biotek – 8000, USA) ELISA plate reader. The survival percentage was calculated as follows: Cell survival percentage = (OD of test-treated cells / OD of control-treated cells) X 100. Results are expressed as the mean percent survival ± SD.

### DNA extraction, whole-genome sequencing, assembly, and annotation

The candidate isolates were streaked on MRS agar plates and incubated at 37 °C under microaerophilic conditions for 48 h. Overnights of the isolated strains were centrifuged at 13,000 × g for 2 min. to pellet the cells. The supernatant was removed, and the pellets were subsequently incubated with 100 μL of Lysozyme (10 mg/mL) (Bio Basic Inc., Canada) for 1 h at 37 °C, and then the total DNA was extracted using the GeneJET Genomic DNA Purification Kit (Thermo Scientific, USA) according to the reference manual. The DNA was reconstituted in 50 µl nuclease free water and stored at -20 ºC. The concentration and quality of the DNA were measured using a NanoDrop ND-1000 spectrophotometer (Thermo Fisher Scientific, USA) [29]. High purity genomic DNA expressing an OD260/OD280 ratio of 1.8–2.0 was used for whole-genome sequencing [30]. The DNA sequencing was performed by Admera Health (New Jersey, USA). The integrity of the genomic DNA was visualized using 1% agarose gel electrophoresis in 0.5 × TBE buffer. DNA libraries were prepared using KAPA HyperPrep Minimal PCR Kit (Roche, USA) according to the manufacturer’s protocol. Whole-genome sequencing was performed on the NovaSeq platform (Illumina, California, USA). Raw data from the Illumina sequencing were cleaned by removing the reads with low quality (< 20) or adapter contamination using Trimmomatic (version 0.38) [31]. Subsequent genomics assembly was performed with all sequencing data using SPAdes (Version 3.12.0) software [32] on the Galaxy Europe server (https://usegalaxy.eu/) [33]. The assessment of the assembled genomes’ quality was performed using QUAST (version 5.2.0) [34]. The two assemblies sequences were deposited in NCBI under submission numbers SUB11934371 (*E. lactis* 10NA) and SUB11931917 (*E. lactis* 10NA). The final assembled genomes were assessed for their degree of completeness and the presence of contamination using checkM (version 1.2.0) [35]. Subsequently, the final assembled genomes were annotated using the Prokaryotic Genome Annotation Pipeline (PGAP) algorithm (NCBI, Bethesda, MD, USA) [36]. The functional characterization of annotated proteins was carried out using the COG functional category through the utilization of eggNOG-mapper software [37, 38]. The antiSMASH 7.0 tool was employed to identify and analyze gene clusters associated with secondary metabolites biosynthesis [39].

### Identification using the whole genome sequence

The average nucleotide identity (ANI) was calculated using fastANI [40]. Identity was confirmed using the Taxonomy-Check module in PGAP [41]. An ANI > 95% represents the same bacterial species. The Type (Strain) Genome Server (TYGS, https://tygs.dsmz.de) [42] and Ribosomal Multilocus Sequence Typing (rMLST, https://pubmlst.org/bigsdb?db=pubmlst_rmlst_seqdef_kiosk) [43] with whole genome sequences input were also used for species identification of the isolates.

### Determination of virulence factors and antibiotic resistance genes (ARGs)

The presence of virulence factors and toxin genes in the isolates genomes was searched using the virulence factor database (VFDB) [44] (last updated: Sep 1, 2023), available at http://www.mgc.ac.cn/cgi-bin/VFs/v5/main.cgi. In addition, the GhostKOALA search tool in the Kyoto Encyclopedia of Genes and Genomes (KEGG) database [45] (Release 107.1), available at https://www.kegg.jp/, was used and inspected for virulence factors and undesirable genes.

The genetic determinants conferring antibiotic resistance in the genome were searched using two publicly available databases: the Comprehensive Antibiotic Resistance Database (CARD) available at https://card.mcmaster.ca/ [46] and the KEGG database (Release 107.1) using the GhostKOALA search tool and inspected under “Brite ko01504: Antimicrobial resistance genes” [45]. The potential transferability of the ARGs identified in the two genomes was explored by assessing their positions in relation to the identified mobile elements, such as plasmids, and prophages. Regarding IS elements, we conducted a correlation analysis between the presence of all unique IS elements identified in our two genomes and the presence of certain virulence or ARGs [47, 48] in different *E. lactis* and *E. faecium* strains (Table S1). The IS16 element was utilized as a positive reference for assessing pathogenicity correlation [49].

### Characterization of putative mobile genetic elements and potential defense systems

Potential plasmid sequences were extracted and classified from the draft assemblies by employing the MOB-suite software tools [50] which were accessible via the Galaxy Europe server (https://usegalaxy.eu/) [33]. Putative prophage sequences in the isolates were detected using PHASTER (https://phaster.ca/) [51]. Bacterial insertion sequences (ISs) were identified using ISfinder [52]. Regarding possible defense systems, clustered regularly interspaced short palindromic regions (CRISPR) were detected through the use of the CRISPRCasFinder tools [53]. Additionally, we explored the presence of restriction-modification (RM) enzymes in our two genomes using Restriction-ModificationFinder 1.1 available at https://cge.food.dtu.dk/services/Restriction-ModificationFinder/.

### Dataset selection for in silico comparative genomics analyses

The dataset used in our study was selected using the NCBI Reference Sequences (RefSeq) database [54] (accessed on August 15, 2023). A total of 227 of the latest RefSeq assemblies for *E. lactis* species were available. The comparative analyses was conducted using closely related *E. faecium* species. For the selection of *E. faecium* isolates, distinct sets were generated using specific keywords within the biosample database, categorizing them into potential pathogenic, potential nonpathogenic, and potential probiotic *E. faecium* strains. In order to reduce the large number of obtained *E. faecium* biosamples and ensure representation of a wide genomic range, we implemented a geographic location filter for both the potential pathogenic and potential nonpathogenic *E. faecium* datasets. Within this filter, a single isolate was randomly chosen from each geographical location. Notably, taxonomic misclassifications were detected between *E. lactis* and *E. faecium* species [55]. To address this concern, genomes exhibiting Average Nucleotide Identity (ANI) values less than 95% within each species were excluded from the final selected dataset. Furthermore, to decrease redundancy, genomes displaying ANI values exceeding 99.9% were also omitted [56]. The comprehensive selected dataset, along with all relevant metadata, is available in Table S1. Ultimately, our selected dataset comprised 198 genomes, with 76 genomes belonging to the *E. faecium* species and 122 genomes to the *E. lactis* species.

### Investigating probiotic and safety characteristics of *E. lactis* species using comparative in silico analyses

In order to investigate the probiotic potential within the *E. lactis* species, we conducted Principal Component Analysis (PCA) using homologous genes associated with probiotic activity. These genes were collected from existing literature [57, 58] and various databases. Specifically, the KEGG database was utilized to acquire genes involved in the synthesis pathways of essential amino acids such as Threonine, Methionine, Valine, Isoleucine, Leucine, Lysine, Histidine, Tryptophan, and Phenylalanine. Additionally, genes responsible for the synthesis of various vitamins, including Riboflavin, Thiamine, Pantothenate, Tetrahydrofolate, Biotin, Menaquinone, Pyridoxal, Coenzyme-A, and Cobalamin, were retrieved. Furthermore, we assessed the presence of potential antimicrobial peptides by using the dbAMP database (Version 2.0) [59, 60] available at https://awi.cuhk.edu.cn/dbAMP/. Additionally, our analysis incorporated virulence genes and ARGs present in virulent *E. faecium* strains [47, 48].

For evaluating the potential safety of *E. lactis* species, we employed the selected *Enterococcus* strains mentioned previously in a phylogenetic analysis. Core genome proteins were extracted using Roary [61], and the phylogenetic analysis with these core genome proteins was conducted using PhyloPhlAn 3.0 [62]. PCA was performed on the shell genes present in 15% to 95% of the strains retrieved by Roary for all *E. lactis* strains and representative *E. faecium* strains, serving to assess the overall safety profile of the *E. lactis* species.

### Statistical analyses and visualization

Statistical analyses were performed using R software (version 4.3.1). The data presented here is expressed as the arithmetic mean of three repetitions ± SD. A student’s t-test was used to test for a significant difference from the control in the acid resistance experiment. On the other hand, a one-way ANOVA was used for the bile resistance significant analysis. In the cytotoxicity assay, a two-way ANOVA was carried out to test the effect of supernatant source and concentration on Caco-2 survivability. Principal Component Analysis (PCA) was performed using the FactoMineR and factoextra packages. The calculation of Spearman correlations between IS elements and virulence factors or ARGs was carried out utilizing the stats package, and the corresponding figures were generated through the ggplot2 packages. Visualization of the phylogenetic tree was achieved using the ggtree package.

For the two *Enterococcus lactis* strains used in this study, their genome assemblies were submitted to the National Center for Biotechnology Information (NCBI) database. GenBank accession numbers for *E. lactis* 10NA and 50NA strains are JANQBF000000000 and JANQBE000000000, respectively. All genome sequences used in this study are publicly available in the NCBI database.

## Results

### Isolation and examination of potential probiotic properties

**Fig. 1:**
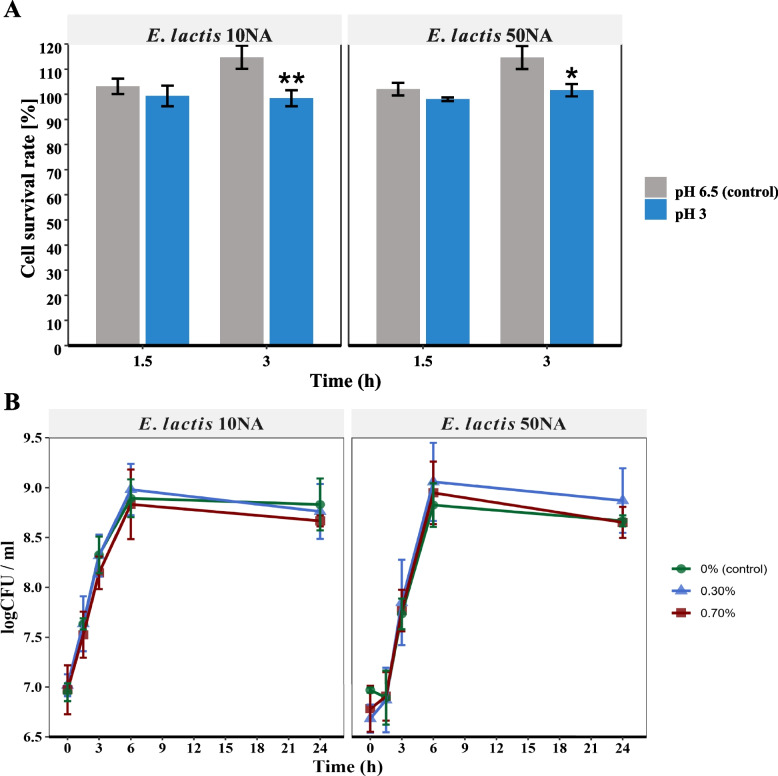
Assessment of the probiotic properties of *E. lactis* 10NA and *E. lactis* 50NA strains. **A** Acid survivability percent of the two strains in MRS media with pH adjusted at 3; the control was plain MRS media with pH 6.5. **B** Growth curves (log CFU/ml) of the two strains in MRS media with 0% (control), 0.3%, and 0.7% (w/v) bile salts concentrations. The values are presented as the means ± SD of three independent experiments. “*” indicates a significant difference where “*”*P*-value < 0.05 and “**”*P*-value < 0.01

Distinct non-hemolytic colonies were selected and examined under a microscope. Two Gram-positive enterococci strains were chosen for further investigation. In assessing acid resistance, it was observed that both strains displayed remarkable resilience in acidic conditions. Despite significant differences (*P* < 0.01 and *P* < 0.05) noted between the 10NA and 50NA strains compared to the control after 3 h, their survival rate remained above 98% during 3 h of incubation at pH 3 (Fig. 1A). Analyzing the growth curve of the two strains in MRS broth (control) and MRS broth supplemented with bile salt concentrations of 0.3% (w/v) and 0.7% (w/v), both strains exhibited normal growth in the presence of bile salts up to 0.7% (w/v) (Fig. 1B), with no significant differences (*P* > 0.05) compared to the control at any time point. Both of our selected strains exhibited inhibitory effects on the growth of *Clostridioides difficile*, *Salmonella typhi*, and *Escherichia coli*. The antimicrobial activity was observed as a clear zone within the overlay layer containing the pathogenic strains.

### Phenotypic safety assessment

#### Antibiotic susceptibility of the isolated strains

The two strains displayed different antibiotic susceptibility profiles (Table 1). Both were highly sensitive to vancomycin, tetracycline, and chloramphenicol. While both isolates showed resistance to kanamycin. Strain 50NA was sensitive to clindamycin, while strain 10NA was moderately sensitive. Moreover, strain 50NA was sensitive to erythromycin and moderately sensitive to gentamicin and streptomycin. On the other hand, strain 10NA was resistant to erythromycin, gentamicin, and streptomycin.

**Table 1:** Susceptibility of the tested strains to antibiotics using the disc diffusion method

Antibiotics	Inhibition zone diameter in mm (mean ± SD)	
	**10NA (*E. lactis*)**	**50NA (*E. lactis*)**
**Chloramphenicol (30 μg/disc)**	25 ± 0.58	24 ± 2.08
	S	S
**Clindamycin (2 μg/disc)**	16 ± 0.58	25 ± 1.15
	I	S
**Erythromycin (15 μg/disc)**	10 ± 1.73	23 ± 2.65
	R	S
**Gentamicin (10 μg/disc)**	11 ± 1	14 ± 0.58
	R	I
**Kanamycin (30 μg/disc)**	0 ± 0	0 ± 0
	R	R
**Streptomycin (10 μg/disc)**	10 ± 0.58	13 ± 1
	R	I
**Tetracycline (30 μg/disc)**	26 ± 0.58	27 ± 0.58
	S	S
**Vancomycin (30 μg/disc)**	20 ± 2.08	21 ± 1.73
	S	S

*R* Resistant, *I* Intermediate, *S* Susceptible, according to the breakpoints established by the CLSI

#### Cytotoxicity/anti-proliferative activity of the isolated strains’ CFSs towards Caco-2 cells

**Fig. 2:**
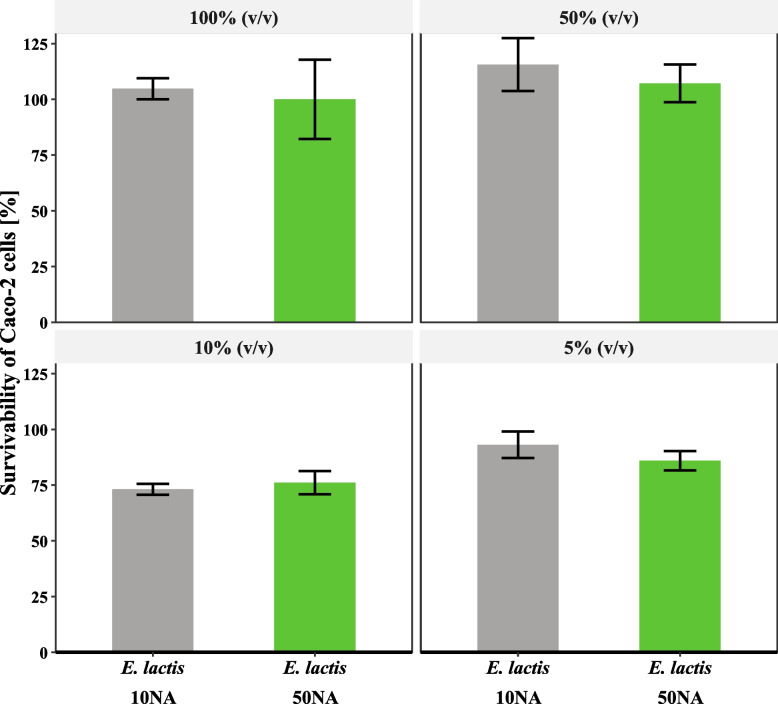
Cytotoxicity assay of *E. lactis* 10NA and *E. lactis* 50NA strains’ supernatants on Caco-2 cells. Survivability percent of Caco-2 cells relative to control in the bacterial culture supernatants with different concentrations (100% (v/v), 50% (v/v), 10% (v/v), and 5% (v/v)). The control used is sterile MRS broth with the same concentrations as the tested supernatants. The values are presented as the mean ± SD of three replicates

The survival of Caco-2 cells in the presence of 4 different concentrations of each isolate CFS was analyzed (Fig. 2). Overall, the survival rate of Caco-2 did not show any significant difference (*P* > 0.05) between the control supernatant and any of the two strains supernatants. While the concentration factor affected survival, with 10% (v/v) concentration having the lowest survival percentage (*P* < 0.05). In general, the two strains’ CFSs did not possess anti-proliferative activity against human colon Caco-2 cells.

### Genomic features and annotations of the two assembled genomes

**Fig. 3:**
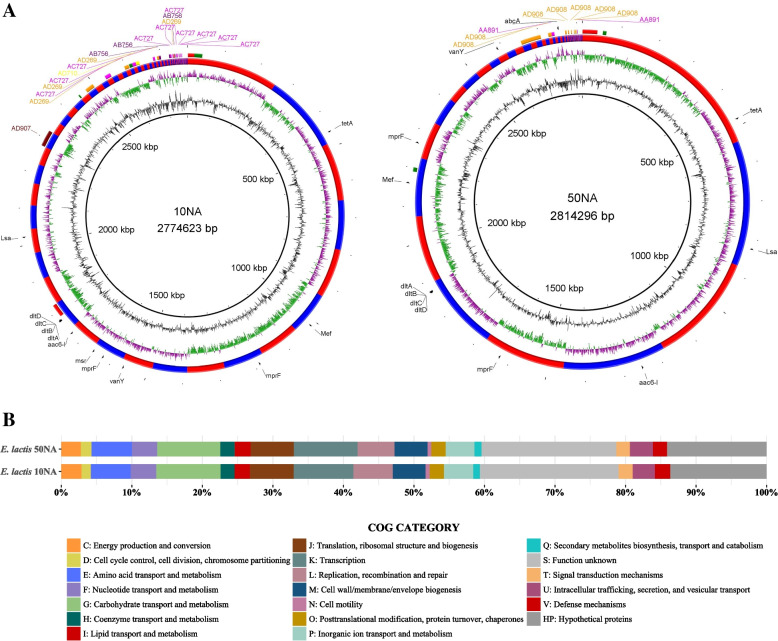
Genomic features and annotations of *E. lactis* 10NA and *E. lactis* 50NA strains. **A** Representation of the genomes of both strains. Circular layers, moving from the innermost to the outermost: GC content (depicted in black), GC Skew (illustrated in green and purple), demarcation of contig boundaries (shown in alternating red and blue), mobile genetic elements (including plasmids* and prophages**), and ARGs. *Regions with matching colors denote segments within a single plasmid. **The color red designates intact prophages, while green indicates incomplete prophages. **B** Distribution of cog categories in both strains

Total sequences of 2 × 2614879 and 2 × 2670661, with an average quality per read of Q36, were used for the final assemblies of *E. lactis* 10NA and *E. lactis* 50NA, respectively. For *E. lactis* 10NA, the final genome assembly yielded a coverage of 269x, a size of 2,774,623 bp, and a N50 of 106,660 bp. On the other hand, *E. lactis* 50NA exhibited a genome assembly with a coverage of 272x, a size of 2,814,296 bp, and a N50 of 278,172 bp. The completeness percentages for the two *E. lactis* assemblies were 99.13% for 10NA and 99.37% for 50NA. Genomic features for both strains are illustrated in Fig. 3A.

The number of coding genes (CDSs) is 2,559 for *E. lactis* 10NA and 2,593 for *E. lactis* 50NA. Based on the Cluster of Orthologous Group (COG) functional annotation, a total of 1782 (66.7%) CDSs in 10NA and 1805 (66.8%) in 50NA were assigned to 18 COG functional categories (Fig. 3B). However, 525 (19.7%) CDSs and 517 (19.1%) CDSs, in 10NA and 50NA, respectively, were assigned to “S: Function unknown” (Fig. 3B). Moreover, 364 (13.6%) CDSs and 381 (14.1%), in 10NA and 50NA, respectively, had no homologs in the COG database that were designated as “HP: hypothetical proteins” (Fig. 3B). Accordingly, a total of 889 (33.3%) CDSs and 898 (33.2%) CDSs were poorly characterized in both genomes.

**Fig. 4:**
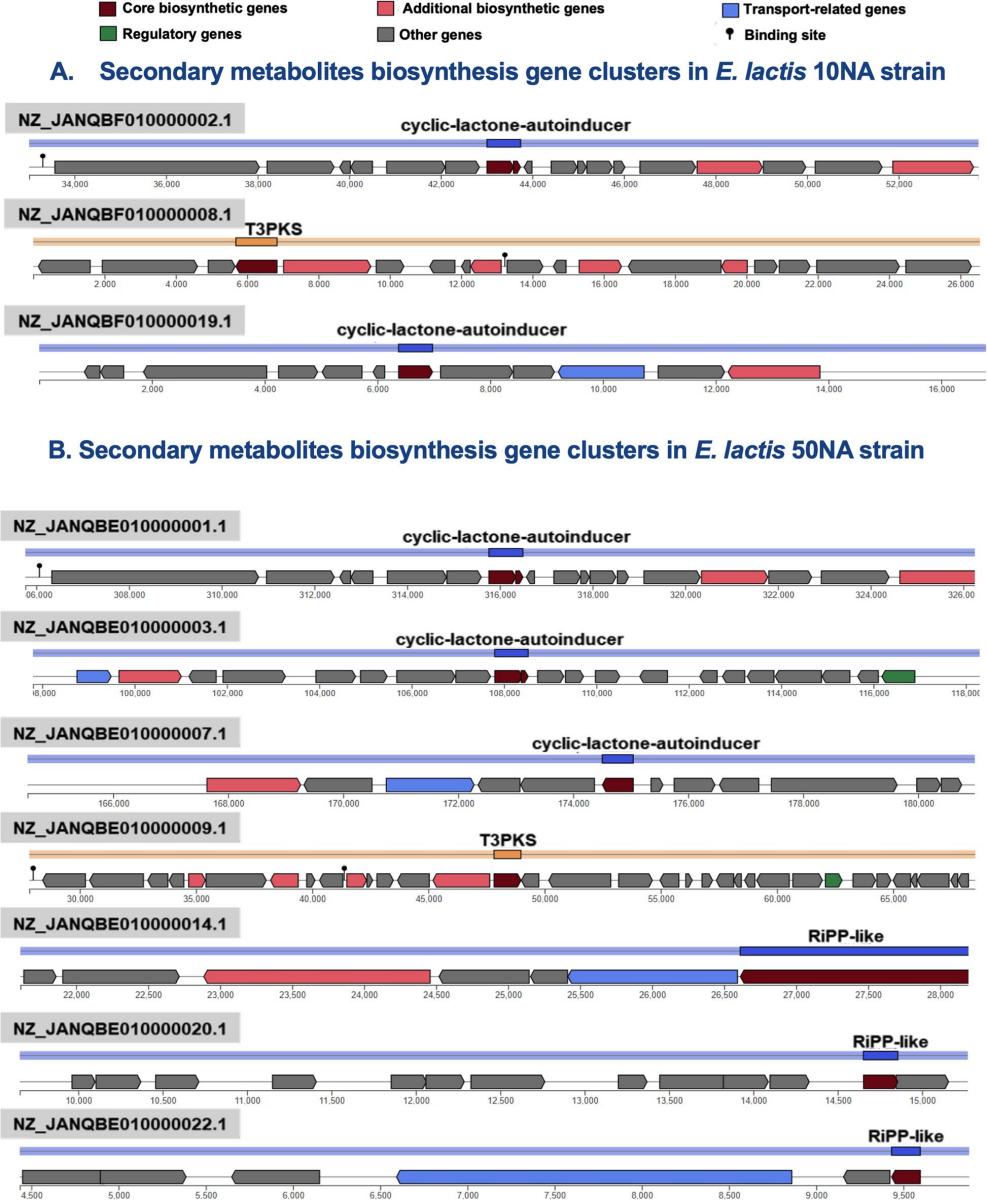
The genetic organization and locations of biosynthetic gene clusters associated with secondary metabolism in both the *E. lactis* 10NA and *E. lactis* 50NA genomes

We have identified three potential biosynthetic gene clusters responsible for secondary metabolite production within the *E. lactis* 10NA strain. Two of these clusters are associated with the synthesis of cyclic lactone autoinducer peptides, while one is involved in Type III PKS (Polyketide synthase) synthesis. On the other hand, the *E. lactis* 50NA strain harbors seven secondary biosynthetic gene clusters. Among these, three are linked to the synthesis of cyclic lactone autoinducer peptides, one is dedicated to Type III PKS, and three are associated with ribosomal synthesized and post-translationally modified peptide products (RiPPs). The organization and positions of these biosynthetic gene clusters within the two genomes are illustrated in Fig. 4.

### Genome-based identification

According to the ANI calculation, the bacterial isolates were assigned to the *Enterococcus lactis* species. Species identification by rMLST was consistent with that by the ANI value, and it was also consistent with that by TYGS. Identification using the 16S rRNA gene sequence gave the best hits with *E. faecium* strains and 100% identity with both *E. faecium* and *E. lactis* strains.

### Genotypic safety assessment of the two isolated strains

#### Determination of virulence factors and undesirable genes

VFDB is generally known for its low specificity, often returning genes that are essential for bacterial survival and competition (Table S2). To identify virulence genes, we turned to the KEGG database, and by cross-referencing Bacterial toxins under Brite annotations with PGAP annotations, we found that only hemolysin III matched both sets of annotations (Table S3). We also investigated the presence of genes involved in biogenic amine (BA) production and D-lactic acid production. The KEGG database proved to be efficient for this search, as it includes enzymes related to these significant BA production pathways and D-lactic acid production. We did not find any genes associated with the production of biogenic amines such as cadaverine, putrescine, spermidine, spermine, ornithine, histamine, and tryptamine in the genomes of both isolates. The only gene related to BA production that we detected was the tyrosine decarboxylase gene responsible for tyramine production (Table S3). Regarding D-lactic acid production, there are two reported genes involved in this pathway: lactate racemase and D-lactate dehydrogenase. However, only the D-lactate dehydrogenase gene was identified in both the 10NA and 50NA genomes. Therefore, it appears that the pathway for D-lactic acid production is incomplete in both isolates.

#### Antimicrobial resistance genes (ARGs)

The analysis was done using two ARGS databases, namely CARD and KEGG. In the case of *E. lactis* 10NA, the CARD database yielded 2 strict matches and 161 loose matches. For *E. lactis* 50NA, there were 2 strict matches and 164 loose matches in CARD. The strict hits in both genomes were: AAC(6')-Ii, responsible for aminoglycoside antibiotic resistance, and vanY, which confers resistance to glycopeptide antibiotics. The KEGG database, in contrast, exhibited greater specificity and sensitivity, identifying a total of 11 potential ARGs in the genomes of both isolates. Notably, the two strict hits initially identified by CARD were also included within the ARGs retrieved from the KEGG database (Fig. 3A and Table S4).

#### Mobile genetic elements identification and potential defense systems detection

Utilizing the MOB-Recon tool for plasmid typing in both strains, five potential plasmids were successfully assembled from the *E. lactis* 10NA genome. Among these, four were predicted to be non-mobilizable, while one, named AD907, was determined to be conjugative. In contrast, the *E. lactis* 50NA genome yielded two potential non-mobilizable plasmids. The spatial arrangement of these potential plasmids in both strains is visually illustrated in Fig. 3A. Detailed information about these plasmids, including associated metadata, can be found in the supplementary tables (Tables S5A and B). We also conducted an examination of the genomes of these isolates to detect genome-embedded phage genes using the PHASTER program (https://phaster.ca/). Our analysis revealed that both the 10NA and 50NA genomes each harbor two active prophages and additionally, we identified three incomplete prophages in both genomes (Fig. 3A and Table S6). Regarding the identification of IS elements, we identified a total of 14 non-redundant IS elements in the 10NA genome, and the 50NA genome contained 12 non-redundant IS elements. These elements passed the following criteria: an E-value of less than 1e-5, coverage ≥ 60%, and identity ≥ 90%. Further details can be found in Table S7.

In terms of potential defense system detection, we identified a Type I restriction enzyme named LldI in *E. lactis* 10NA, along with its associated methyltransferase. Conversely, in *E. lactis* 50NA, no homologous restriction enzymes of any type were found using the Restriction-ModificationFinder-1.1 Server. However, the genome of the 50NA strain revealed the presence of CRISPR arrays with evidence level 4, along with two cas clusters. In contrast, the 10NA strain had three CRISPR arrays, but they were of weak evidence level 1.

#### Transferability of ARGs and correlation between IS elements presence with undesirable genes

**Fig. 5:**
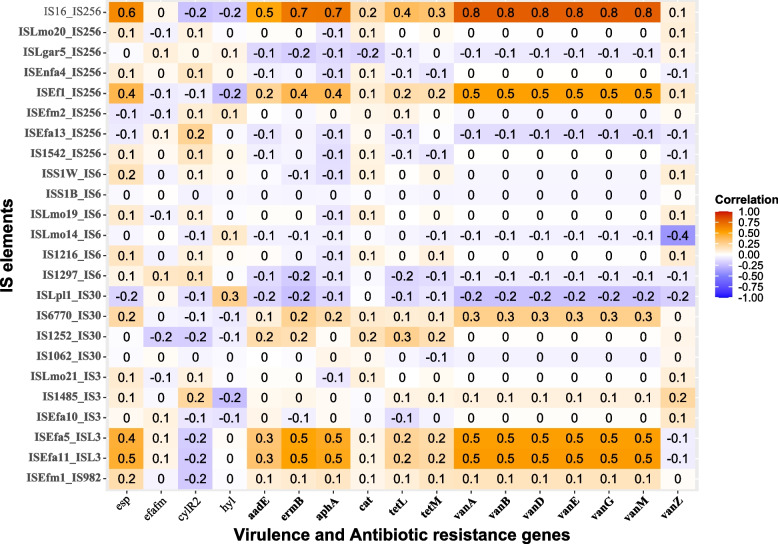
Heatmaps showing the correlation between IS elements and undesirable genes found across different *Enterococcus* genomes. In this representation, the red color indicates a strong positive correlation, while the blue color indicates a strong negative correlation. On the x-axis, the ARGs are denoted by bolded labels, while virulence genes are represented without bolding. Meanwhile, on the y-axis, IS elements identified in our two strains are indicated by bolded labels, with IS16 serving as a positive reference for assessing pathogenicity correlations

None of the identified ARGs were situated within the detected mobile genetic elements, specifically plasmids and prophages, as illustrated in Fig. 3A. Furthermore, we conducted an assessment to determine whether the IS elements present in our genomes could potentially facilitate the horizontal transfer of antibiotic resistance or virulence factors. The results revealed that only a moderate positive correlation (≤ 0.5) existed between the following IS elements: ISEfa11, ISEfa5, and ISEf1 with virulence or ARGs. Conversely, IS16 exhibited a strong correlation (> 0.5) with virulence or ARGs (Fig. 5). Consequently, our conclusion is that the ARGs identified in our two genomes carry a low risk of being transferred to other bacteria. Therefore, our strains do not raise safety concerns in terms of possessing transferable ARGs properties.

### Probiotic features and safety assessment of *E. lactis* species using comparative genomics analysis

**Fig. 6:**
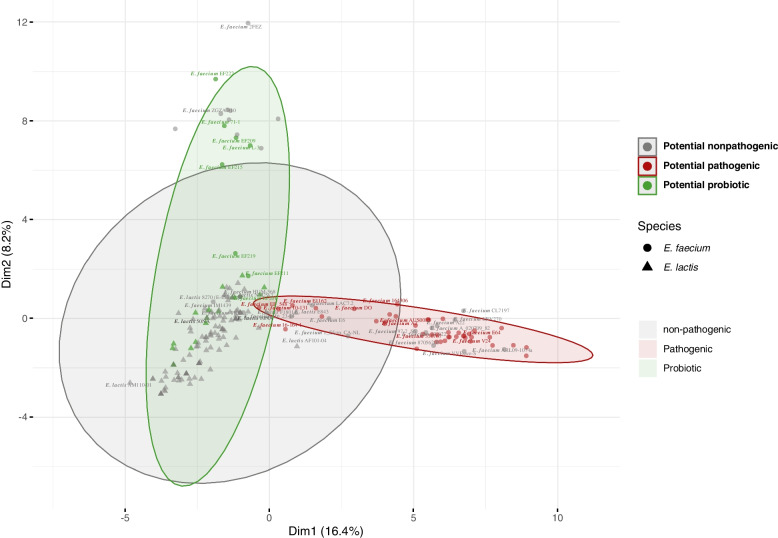
PCA (Principal Component Analysis) of 76 *E. faecium* and 122 *E. lactis* genomes representing potential pathogenic, potential nonpathogenic, and potential probiotic *Enterococcus* strains. The analysis is based on the presence or absence of probiotic genes, essential amino acids and vitamins biosynthesis genes, antimicrobial peptides, virulence factors and antibiotic resistance genes, with a total of 77 genes

We conducted Principal Component Analysis (PCA) on a set of representative *Enterococcus* strains utilizing a comprehensive selection of markers encompassing probiotic, ARGs, and virulence genes, with a total of 77 genes. The results of this analysis delineated distinct clusters for potential probiotic strains and potential pathogenic strains. Almost all *E. lactis* strains clustered within the potential probiotic group, with the exception of *E. lactis* E843, which occupied an outlier position on the periphery of the potential pathogenic cluster. Notably, this particular strain was isolated from swine in China. Among the potential nonpathogenic *E. faecium* strains, approximately half of them clustered within the potential pathogenic group (Fig. 6).

**Fig. 7:**
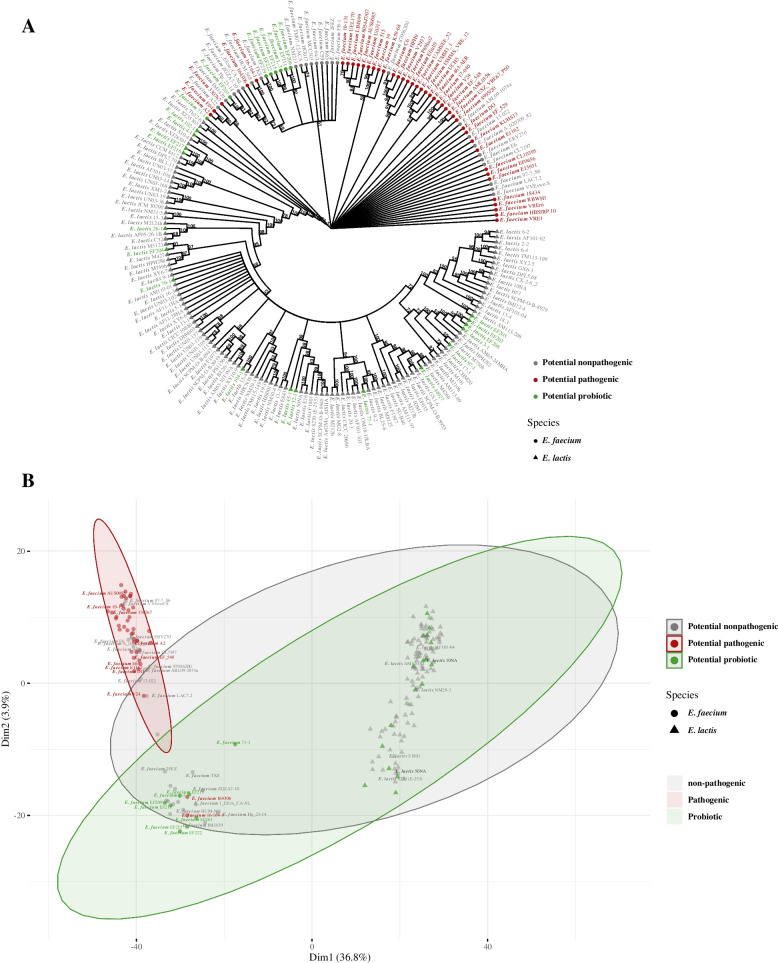
Comparative genomics analysis of 122 *E. lactis* genomes with closely related *E. faecium* 76 representative genomes. **A** Phylogenetic tree of *Enterococcus* genomes sequences based on analysis of 999 core genes. **B** PCA (Principal Component Analysis) for all genomes based on the analysis of 2411 shell genes

Considering the close genetic relatedness between *E. faecium* and *E. lactis* species, we employed a dataset comprising 999 core genome proteins obtained from both species' genomes to construct a phylogenetic tree. This tree confirmed this genetic affinity by clustering most potential pathogenic *E. faecium* strains together, without any *E. lactis* strains within the pathogenic clade (as shown in Fig. 7A). Interestingly, when we performed PCA using the shell genes, encompassing a total of 2411 genes found in both species, it resulted in the formation of a distinct cluster for *E. lactis* strains, with no *E. faecium* strains present within this cluster. Conversely, *E. faecium* strains separated into two distinct clusters, with most potential pathogenic strains forming one cluster that did not include any potential probiotic *E. faecium* strains (Fig. 7B). Notably, two potential pathogenic *E. faecium* strains, 164,306 and 16–164-1, isolated from urinary tract infections, were located in close proximity to potential probiotic *E. faecium* strains. These two strains may not be inherently pathogenic and might not directly contribute to urinary tract infections in affected patients (Fig. 7B). Our findings suggest that *E. lactis* species, in comparison to *E. faecium*, is relatively safe as it lacks pathogenic determinants.

## Discussion

Vast numbers of strains were characterized as potential probiotics. The safety of newly discovered strains must be thoroughly investigated. As guidance for the safety examination, whole genome sequencing provides valuable information for all potential virulence or antibiotic resistance genes. Also, the risk of transferring virulence or resistance to the surrounding environment could be detected. Besides, whole genome analysis is a powerful tool for comparing different strains and species, which allows us to select beneficial, safe strains or species for further investigations [63, 64]. In this study, we isolated two *Enterococcus lactis* strains, 10NA and 50NA, from human stool. These strains showed common probiotic properties such as acid and bile resistance. Antibiotic resistance is not the only shortcoming caused by antibiotic intensive use; ironically, antibiotic administration can cause infections like *Clostridioides difficile* due to microbiota dysbiosis. Therefore, seeking alternatives to antibiotics will decrease emerging resistance and maintain a normal microbiota, which acts as a barrier against notorious pathogens [65, 66]. Antibacterial activity against certain gut pathogens like *E. coli*, *S. typhi* and *C. difficile* was displayed by our two *E. lactis* strains.

For safety evaluation, blood hemolysis is a commonly used test. The two isolated strains did not show any kind of hemolysis on the blood agar. Although some *Lactobacillus* species can develop α- hemolysis or even β-hemolysis, they are used without safety concerns [67, 68]. Functional antibiotic susceptibility of potential probiotics should be determined, and sensitivity towards more than one antibiotic is required. Our two isolated strains were sensitive to commonly used antibiotics as vancomycin, tetracycline, and chloramphenicol. Moreover, the *E. lactis* 50NA strain was sensitive to erythromycin and clindamycin. Overall, the *E. lactis* 50NA strain showed more sensitivity patterns towards antibiotics than *E. lactis* 10NA. On the other hand, the two strains were resistant to kanamycin. Similarly, *E. faecium* SF68, an *Enterococcus* probiotic with a long history of safe use, is resistant to kanamycin [69]. Generally, aminoglycosides resistance is prevalent in lactic acid bacteria (LAB), where the main concern is the transferability of genetic determinants [70, 71]. Regarding the cytotoxicity/anti-proliferative activity of current probiotics, Caco-2 and HT-29 are the most commonly used cell lines [72]. In the present study, the two *E. lactis* strains’ CFSs showed a non-significant difference from the control (*P* > 0.05) overall. While the 10% (v/v) concentration showed the lowest survivability for Caco-2 cells. Similarly, in the study by K Śliżewska, A Chlebicz-Wójcik and A Nowak [67] on *Lactobacillus* strains, only *L. reuteri* ŁOCK 1092 isolate supernatant showed significantly higher anti-proliferative activity than the control at lower concentrations of 5% (v/v) and 10% (v/v). This significant difference disappeared at higher concentrations of 20% (v/v) and 50% (v/v). This may be attributed to the cytotoxic effect of the plain MRS media which blurred the cytotoxicity of the metabolites at higher concentrations.

Whole-genome sequencing for bacterial strains with initial promising probiotic properties and acceptable safety should be done. Long-read sequencing offered by the Oxford Nanopore or PacBio platforms provides complete genomes. However, these sequencing platforms have drawbacks like low accuracy and high cost [73]. The most critical drawback is the inability to detect small plasmids due to the size selection step required for these sequencing techniques [68]. On the other hand, Illumina short-read sequencing has the merits of high accuracy and low cost. But the inability to cover repetitive sequences longer than their reads causes gaps in the final genome assembly. Therefore, hybrid assembly combining short reads and long reads is the best method. Nevertheless, choosing the method for sequencing is a tradeoff between cost and adequacy. Hybrid assemblies are able to differentiate closely related isolates, which is useful in determining the origins of outbreaks and other applications [73]. Since detecting plasmid sequences is critical for the risk evaluation of potential probiotics, Illumina sequencing could provide a cost-effective method for investigating potential probiotics. In this study, whole genome assemblies of coverage ~ 270 × for our two isolates were achieved using Illumina short-read sequencing. Whole genomic based identifications were inconsistent with 16S RNA gene identification, which emphasize the necessity of whole genome sequencing for precise identification of bacterial isolates. The accurate taxonomic placement is crucial for the identification of possible risks associated with the taxon. Analyzing secondary metabolites within bacterial genomes provides insight into the genotypic factors responsible for various bacterial applications, including their potential as probiotics or for biotechnological purposes. In the case of our strains, the 10NA strain is equipped with three secondary biosynthetic gene clusters, while the 50NA strain harbors seven such clusters. These secondary metabolites primarily fall into categories such as cyclic lactone autoinducer peptides and Type III PKS (Polyketide synthase) synthesis. Conversely, in the case of E. lactis 50NA, its secondary metabolites encompass cyclic lactone autoinducer peptides, Type III PKS, as well as ribosomal synthesized and post-translationally modified peptide products (RiPPs). Cyclic lactone autoinducer peptides play a pivotal role in bacterial communication [74]. Additionally, RiPPs exhibit antibacterial activity against various pathogens [75], a trait that is phenotypically expressed in both of our strains. These secondary metabolites enhance the probiotic potential of the selected strains. In the search for all possible virulence factors (VF) and antibiotic resistance (AR) genes, our two *E. lactis* genomes were blasted against the VFDB and CARD databases. Linking the retrieved genetic AR determinants to the observed phenotypic resistance patterns of the two strains, the AAC(6')-Ii gene was present in the two strains’ genomes which could account for intrinsic aminoglycoside resistance, including kanamycin [70]. Also, the msr gene responsible for macrolide resistance was present in the 10NA genome but not in the 50NA genome (Table S4). This could explain the resistance of the 10NA strain to erythromycin, to which the 50NA strain was sensitive. The next step was to predict the risk posed by these genes. Nevertheless, most virulence factors retrieved by VFDB are required for bacterial survival and competition. Together with their intrinsic resistance to some antibiotics, potential probiotics can survive in competitive environments. All mobile genetic elements detected in the two genomes, including plasmids, and prophages, did not harbor any VF or AR genes. However, among the five potential plasmids detected in the *E. lactis* 10NA strain, one is predicted to be conjugative, whereas the two potential plasmids in the 50NA strain were non-mobilizable. Although neither of our two genomes carries the IS16 insertion element, which is a predictive marker for pathogenic *E. faecium* strains [49], we confirmed the safety of the IS elements found in the two *E. lactis* strains through correlation analysis. None of the identified IS elements displayed a strong correlation with known virulence factors or resistance genes. Previous studies in the field of *Enterococcus* probiotics focused on the lack of transferable virulence factors and antibiotic resistance genes. However, these safe strains might accept transferable elements from other pathogenic strains. This dilemma is the main reason for the reluctance to approve *Enterococcus* as a safe probiotic supplement. Accordingly, the presence of defenses that prevent the acceptance of foreign transferable elements could ameliorate the problem. Prokaryotes employ a variety of defense mechanisms, with the most prevalent ones being restriction-modification (RM) systems and CRISPR–Cas immune systems [13]. RM systems function by cleaving foreign DNA, serving as an innate defense mechanism. In contrast, the CRISPR-Cas system has garnered significant attention due to its capacity to manipulate bacterial genetic elements and its potential associations with antibiotic resistance genes (ARGs) in various species [76–78]. However, it's important to note that these defense mechanisms do not entirely block horizontal gene transfer; instead, they selectively inhibit the uptake of foreign genetic elements [13, 79]. Notably, among nosocomial pathogens like *E. faecium* and *E. faecalis*, the absence of the CRISPR-Cas system appears to enhance their ability to acquire ARGs [16, 80]. Understanding the restriction-modification defense mechanisms in *Enterococcus* species remains a subject in need of further exploration [81]. It's worth mentioning that a complete CRISPR-Cas system was detected in the *E. lactis* 50NA strain, while the 10NA strain exhibited RM type I. Additionally, we conducted Principal Component Analysis (PCA) using probiotic, virulence, and ARGs on a dataset comprising 198 *Enterococcus* genomes. Consistent with previous studies [63, 64], potential pathogenic strains were clearly distinguished from potential nonpathogenic and potential probiotic strains within the analysis.

Despite the rise in enterococcal infections, no infections were associated with *E. lactis* species. In addition to scientific evidence suggesting pathogenic *E. faecium* possessing distinct genomic features, non-pathogenic *E. faecium* clade B strains were phylogenetically belonging to *E. lactis* species [55, 82]. In order to validate the safety of *E. lactis* species, we performed phylogenetic analysis for selected set of *E. lactis* genomes on NCBI in addition to representative genomes for *E. faecium* using 999 core genes present in all genomes. In addition, the shell genes present in 15% to 95% of the selected *Enterococcus* genomes were used in the PCA. Our results showed that potential pathogenic *E. faecium* strains formed a distinct phylogenetic clade. Moreover, the potential pathogenic *E. faecium* strains formed a distinct cluster in the PCA. In both analyses, neither of the *E. lactis* strains was related to the pathogenic *E. faecium* strains.

## Conclusion

A phenotypic assessment of the two isolated *E. lactis* strains from human stool manifested potential probiotic candidates. Complementation with whole genome analysis revealed the lack of transferable virulence factors or antibiotic resistance genes. A complete CRISPR-cas system was discovered in the *E. lactis* 50NA strain, which could decrease predisposition to ARGs acquisition. None of the IS elements present in the two strains were strongly correlated with any undesirable genes. Secondary metabolites belonging to cyclic lactone autoinducer peptides, Type III PKS, and RiPPs were detected in our two genomes. The comparative genomics analysis confirmed the clustering of the two strains with potential probiotics and potential nonpathogenic *Enterococcus* strains. Phylogenetic analysis and principal component analysis provided evidence for the safety of *E. lactis* species. In sum, our study provides compelling evidence for the safety of the two isolated *E. lactis* strains, 10NA and 50NA, and supports the potential use of *E. lactis* species as a source for *Enterococcus*-based probiotics. These findings have important implications for the development and application of probiotics in the context of microbiome research and human health.

### Supplementary Information

**Additional file 1: Supplementary Figure S1.** The fecal samples, age distribution is shown in Figure S1-A, and gender distribution is shown in Figure S1-B.

**Additional file 2: Table S1.** List of Enterococcus strains used in all in silico analyses with their associated metadata. **Table S2A.** List of known and predicted virulence factors* with best hits for Enterococcus genus in E. lactis 10NA genome.** **Table S3A.** List of bacterial toxins / toxic metabolites genes* and their locations in E. lactis 10NA genome. **Table S3B.** List of bacterial toxins / toxic metabolites genes* and their locations in E. lactis 50NA genome. **Table S4A.** List of antibiotic resistance genes* and their locations in E. lactis 10NA genome. **Table S4B.** List of antibiotic resistance genes* and their locations in E. lactis 50NA genome. **Table S5A.** List of Plasmids* and their metadata in E. lactis 10NA genome. **Table S5B.** List of Plasmids* and their metadata in E. lactis 50NA genome. **Table S6A.** List of prophages* and their locations in E. lactis 10NA genome genome. **Table S6B.** List of prophages* and their locations in E. lactis 50NA genome genome.

## Data Availability

For the two *Enterococcus* lactis strains used in this study, their genome assemblies were submitted to the National Center for Biotechnology Information (NCBI) database. GenBank accession numbers for *E. lactis* 10NA and 50NA strains are JANQBF000000000 and JANQBE000000000 respectively. All genome sequences used in this study are publicly available in the NCBI database. All genes related to virulence factors, antibiotic resistance, toxins or toxic metabolites with their metadata and mobile genetic elements with their locations and sizes are listed. All these data are documented in the supplementary file.
