# Supplementary material for: Safety assessment of Enterococcus lactis strains complemented with comparative genomics analysis reveals probiotic and safety characteristics of the entire species
Source: BMC Genomics. 2023 Nov 6;24:667. doi: 10.1186/s12864-023-09749-9 (PMC10626658; doi:10.1186/s12864-023-09749-9)
Supplement: Supplementary file 1 — Additional file 1: Supplementary Figure S1. The fecal samples, age distribution is shown in Figure S1-A, and gender distribution is shown in Figure S1-B. [file 12864_2023_9749_MOESM1_ESM.pdf]

Safety assessment of *Enterococcus lactis* strains complemented with comparative genomic analysis reveals probiotic and safety characteristics of the entire species

Noha A. Ahmed*[1], Rania Abdelmonem Khattab[1], Yasser M. Ragab[1], Mariam Hassan*[1,2]

[1]*Microbiology and Immunology Department, Faculty of Pharmacy, Cairo University, Kasr Al-Aini 11562, Cairo, Egypt.*
[2]*Department of Microbiology and Immunology, Faculty of Pharmacy, Galala University, New Galala City, 43511 Suez, Egypt.*
***Corresponding authors:**
Mariam Hassan, Tel.: +20(122)3376326, Address: Faculty of Pharmacy Cairo University - Kasr El-Aini Street – 11562 - Cairo, Egypt, mariam.hassan@pharma.cu.edu.eg
Noha A. Ahmed, Tel.: +20(100)7910848, Address: Faculty of Pharmacy Cairo University - Kasr El-Aini Street – 11562 - Cairo, Egypt, noha.adel@pharma.cu.edu.eg

Supplementary Figure S1

The fecal samples, age distribution is shown in Figure S1-A, and gender distribution is shown in Figure S1-B.

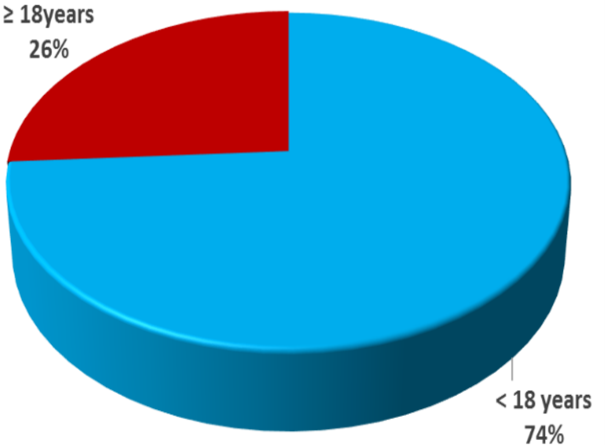

Figure S1-A: Age distribution of the fecal samples.

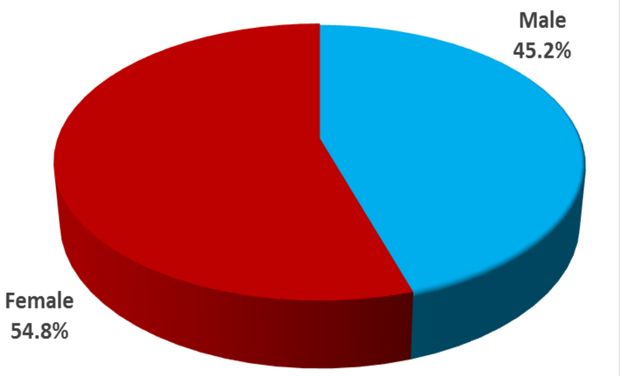

Figure S1-B: Gender distribution of the fecal samples.
