# Supplementary material for: Safety assessment of Enterococcus lactis strains complemented with comparative genomics analysis reveals probiotic and safety characteristics of the entire species
Source: BMC Genomics. 2023 Nov 6;24:667. doi: 10.1186/s12864-023-09749-9 (PMC10626658; doi:10.1186/s12864-023-09749-9)
Supplement: Supplementary file 2 — Additional file 2: Table S1. List of Enterococcus strains used in all in silico analyses with their associated metadata. Table S2A. List of known and predicted virulence factors* with best hits for Enterococcus genus in E. lactis 10NA genome.** Table S3A. List of bacterial toxins / toxic metabolites genes* and their locations in E. lactis 10NA genome. Table S3B. List of bacterial toxins / toxic metabolites genes* and their locations in E. lactis 50NA genome. Table S4A. List of antibiotic resistance genes* and their locations in E. lactis 10NA genome. Table S4B. List of antibiotic resistance genes* and their locations in E. lactis 50NA genome. Table S5A. List of Plasmids* and their metadata in E. lactis 10NA genome. Table S5B. List of Plasmids* and their metadata in E. lactis 50NA genome. Table S6A. List of prophages* and their locations in E. lactis 10NA genome genome. Table S6B. List of prophages* and their locations in E. lactis 50NA genome genome. [file 12864_2023_9749_MOESM2_ESM.pdf]

Safety assessment of *Enterococcus lactis* strains complemented with comparative genomic analysis reveals probiotic and safety characteristics of the entire species

Noha A. Ahmed*1, Rania Abdelmonem Khattab1, Yasser M. Ragab1, Mariam Hassan*1,2

1Microbiology and Immunology Department, Faculty of Pharmacy, Cairo University, Kasr Al-Aini 11562, Cairo, Egypt.

2Department of Microbiology and Immunology, Faculty of Pharmacy, Galala University, New Galala City, 43511 Suez, Egypt."

Table S1. List of *Enterococcus* strains used in all in silico analyses with their associated metadata.

Biosample accession	Strain	Geographic location	Host	Host disease	Isolation	RefSeq assembly accession	Predicted phenotype
SAMN06111311	E. faecium 1_Efcm_CA-NL	Netherlands	Homo sapiens	-	feces	GCF_002442445.1	Potential nonpathogenic
SAMN08149825	E. faecium 13-022	France: Caen	Homo sapiens	-	feces	GCF_002997315.1	Potential nonpathogenic
SAMN13155884	E. faecium 2FEZ	Portugal: Matosinhos	Homo sapiens	-	feces from hospitalized patient	GCF_009707525.1	Potential nonpathogenic
SAMN04009964	E. faecium 64/3	Germany	Homo sapiens	-	stool sample	GCF_001298485.1	Potential nonpathogenic
SAMN04266627	E. faecium 87056200	Sweden: Gaevle	Homo sapiens	-	feces	GCF_001542895.1	Potential nonpathogenic
SAMN06133059	E. faecium 97-7_S6	Chile	Homo sapiens	-	feces	GCF_001990615.1	Potential nonpathogenic
SAMN06006823	E. faecium A_020709_82	Netherlands: University Medical Centre Utrecht	Homo sapiens	-	feces	GCF_002442955.1	Potential nonpathogenic
SAMN09466876	E. faecium A22	Netherlands: Groningen	Homo sapiens	-	feces	GCF_003268235.1	Potential nonpathogenic
SAMN09435330	E. faecium ARL09-1074a	New Zealand	Homo sapiens	-	feces	GCF_005234895.1	Potential nonpathogenic
SAMN13155882	E. faecium C59	Portugal: Porto	Homo sapiens	-	feces from healthy volunteer	GCF_009707505.1	Potential nonpathogenic
SAMN19459754	E. faecium CL7197	Brazil	Homo sapiens	-	feces	GCF_020103675.1	Potential nonpathogenic
SAMN06020035	E. faecium E6	Spain: Madrid	Homo sapiens	-	feces	GCF_001953455.1	Potential nonpathogenic
SAMN05461971	E. faecium ERV270	Colombia: Bogota	Homo sapiens	-	feces	GCF_008131615.1	Potential nonpathogenic
SAMN11974796	E. faecium FB-1	South Korea	Homo sapiens	-	feces	GCF_006351785.1	Potential nonpathogenic
SAMN30076183	E. faecium HG017	India: Pune	Homo sapiens	-	feces	GCF_024723285.1	Potential nonpathogenic
SAMN07728629	E. faecium Hp_23-14	Russia: Kazan	Homo sapiens	-	feces from HP_010	GCF_002631325.1	Potential nonpathogenic
SAMN15957561	E. faecium HUM-568	Estonia: Tartu	Homo sapiens	-	feces	GCF_014853835.1	Potential nonpathogenic
SAMEA11998744	E. faecium IM1439	Serbia	-	-	traditional Serbian cheese	GCF_925297835.1	Potential nonpathogenic
SAMN14389594	E. faecium K65	China	Homo sapiens	-	feces	GCF_019731045.1	Potential nonpathogenic
SAMN12828129	E. faecium LAC7.2	Brazil: Parana	-	-	Oreochromis niloticus gut	GCF_009036045.1	Potential nonpathogenic
SAMN12147396	E. faecium MCC503	Ireland	Homo sapiens	-	feces	GCF_018784845.1	Potential nonpathogenic
SAMN10697318	E. faecium NM213	Egypt:Cairo	Homo sapiens	-	infant feces	GCF_005166365.1	Potential nonpathogenic
SAMN33443761	E. faecium TK8	Algeria	-	-	milk	GCF_028993955.1	Potential nonpathogenic
SAMN31809866	E. faecium TM07-12ACA	China: Shenzhen	Homo sapiens	-	fecal material	GCF_027689725.1	Potential nonpathogenic
SAMN12138986	E. faecium VVEswe-S	Sweden:Halmstad	Homo sapiens	-	feces	GCF_007917315.3	Potential nonpathogenic
SAMN22155556	E. faecium ZGZA7-10	Croatia: Zagorje	-	-	soft fresh cheese	GCF_020514405.1	Potential nonpathogenic
SAMN30311161	E. lactis 10NA*	Egypt	-	-	Homo sapiens stool	GCF_027925905.1	Potential nonpathogenic
SAMN21840361	E. lactis 13-1	China: Hubei	-	-	Rice wine Koji	GCF_029600155.1	Potential nonpathogenic
SAMN21840362	E. lactis 13-3	China: Hubei	-	-	Rice wine Koji	GCF_029600135.1	Potential nonpathogenic
SAMN21840363	E. lactis 13-4	China: Hubei	-	-	Rice wine Koji	GCF_029600115.1	Potential nonpathogenic
SAMN21840377	E. lactis 15-3	China: Hubei	-	-	Rice wine Koji	GCF_029599835.1	Potential nonpathogenic
SAMN21840378	E. lactis 15-4	China: Hubei	-	-	Rice wine Koji	GCF_029599755.1	Potential nonpathogenic
SAMN21840379	E. lactis 16-3	China: Hubei	-	-	Rice wine Koji	GCF_029599805.1	Potential nonpathogenic
SAMN21840380	E. lactis 17-3	China: Hubei	-	-	Rice wine Koji	GCF_029599735.1	Potential nonpathogenic
SAMN21840382	E. lactis 18-3	China: Hubei	-	-	Rice wine Koji	GCF_029599795.1	Potential nonpathogenic
SAMN21840383	E. lactis 19-3	China: Hubei	-	-	Rice wine Koji	GCF_029599665.1	Potential nonpathogenic
SAMN21840385	E. lactis 20-3	China: Hubei	-	-	Rice wine Koji	GCF_029599715.1	Potential nonpathogenic
SAMN21840364	E. lactis 2-2	China: Hubei	-	-	Rice wine Koji	GCF_029600075.1	Potential nonpathogenic
SAMN30311914	E. lactis 50NA*	Egypt	-	-	Homo sapiens stool	GCF_027925885.1	Potential nonpathogenic
SAMN21840366	E. lactis 5-1R	China: Hubei	-	-	Rice wine Koji	GCF_029600055.1	Potential nonpathogenic
SAMN21840369	E. lactis 6-2	China: Hubei	-	-	Rice wine Koji	GCF_029599955.1	Potential nonpathogenic
SAMN21840370	E. lactis 6-4	China: Hubei	-	-	Rice wine Koji	GCF_029599995.1	Potential nonpathogenic
SAMN23828335	E. lactis 8270-(E-253)	Bulgaria: Plovdiv	-	-	milk	GCF_021229185.1	Potential nonpathogenic
SAMN21840373	E. lactis 9-1	China: Hubei	-	-	Rice wine Koji	GCF_029599895.1	Potential nonpathogenic
SAMN21840374	E. lactis 9-2	China: Hubei	-	-	Rice wine Koji	GCF_029599905.1	Potential nonpathogenic
SAMN31807721	E. lactis AF05-26-1B	China: Shenzhen	-	-	Homo sapiens fecal material	GCF_027688035.1	Potential nonpathogenic
SAMN31807757	E. lactis AF101-02	China: Shenzhen	-	-	Homo sapiens fecal material	GCF_027687325.1	Potential nonpathogenic
SAMN31807759	E. lactis AF101-04	China: Shenzhen	-	-	Homo sapiens fecal material	GCF_027687255.1	Potential nonpathogenic
SAMN31807763	E. lactis AF101-103	China: Shenzhen	-	-	Homo sapiens fecal material	GCF_027687175.1	Potential nonpathogenic
SAMN31807764	E. lactis AF101-108	China: Shenzhen	-	-	Homo sapiens fecal material	GCF_027687165.1	Potential nonpathogenic
SAMN31807821	E. lactis AF11-1HA	China: Shenzhen	-	-	Homo sapiens fecal material	GCF_027686015.1	Potential nonpathogenic
SAMN31807824	E. lactis AF11-28HA	China: Shenzhen	-	-	Homo sapiens fecal material	GCF_027685895.1	Potential nonpathogenic
SAMN31808754	E. lactis AM110-01	China: Shenzhen	-	-	Homo sapiens fecal material	GCF_027723505.1	Potential nonpathogenic
SAMN31808994	E. lactis AM113-208	China: Shenzhen	-	-	Homo sapiens fecal material	GCF_027673865.1	Potential nonpathogenic
SAMN31809023	E. lactis AM113-69	China: Shenzhen	-	-	Homo sapiens fecal material	GCF_027672745.1	Potential nonpathogenic
SAMN31809037	E. lactis AM113-97	China: Shenzhen	-	-	fecal material	GCF_027672545.1	Potential nonpathogenic
SAMN31809226	E. lactis AM63-10T3NA	China: Shenzhen	-	-	fecal material	GCF_027667845.1	Potential nonpathogenic
SAMN31809242	E. lactis AM65-24MHA	China: Shenzhen	-	-	fecal material	GCF_027667265.1	Potential nonpathogenic
SAMN17227044	E. lactis AnGM4_AISHA	India:Bengaluru	-	-	goat milk	GCF_016863785.1	Potential nonpathogenic
SAMN21840387	E. lactis BL1-11	Russia: Buryats	-	-	Dairy product	GCF_029599655.1	Potential nonpathogenic
SAMN21840390	E. lactis BL25-6	Russia: Buryats	-	-	Dairy product	GCF_029599495.1	Potential nonpathogenic
SAMN21840388	E. lactis BL26-7	Russia: Buryats	-	-	Dairy product	GCF_029599485.1	Potential nonpathogenic
SAMN35073034	E. lactis C32	China: Beijing	-	-	milk from animal17	GCF_030237875.1	Potential nonpathogenic
SAMD00255145	E. lactis CCM-8412	Italy	-	-	Raw milk Italian cheeses	GCF_015751045.1	Potential nonpathogenic
SAMN31565968	E. lactis CHM20	China	-	-	mesophilic anaerobic digestion	GCF_029733755.1	Potential nonpathogenic
SAMN13420654	E. lactis CICC-10840	missing	-	-	Milk powder	GCF_009735445.1	Potential nonpathogenic
SAMN13426597	E. lactis CICC-20680	missing	-	-	missing	GCF_009735475.1	Potential nonpathogenic
SAMN19908122	E. lactis CX-2-6_2	China:Nanchang	-	-	soil	GCF_019343125.1	Potential nonpathogenic
SAMN22167485	E. lactis DFI.5.68	USA:Chicago	-	-	Homo sapiens fecal sample	GCF_020593395.1	Potential nonpathogenic
SAMN22167494	E. lactis DFI.7.70	USA:Chicago	-	-	Homo sapiens fecal sample	GCF_020593225.1	Potential nonpathogenic
SAMN21366099	E. lactis DH9003	China: Shaan'xi	-	-	goat milk	GCF_020268645.1	Potential nonpathogenic
SAMN16124480	E. lactis E16	Germany	-	-	Surface patient room	GCF_020556885.1	Potential nonpathogenic
SAMN20834202	E. lactis E843	China: Henan	-	-	swine	GCF_019880345.1	Potential nonpathogenic
SAMN29224784	E. lactis EF035	Ghana: Accra	-	-	Rectal swabs	GCF_023956365.1	Potential nonpathogenic
SAMN33901059	E. lactis F1597	Portugal: North and Centre	-	-	chicken feces	GCF_029624325.1	Potential nonpathogenic
SAMN33901069	E. lactis F1801	Portugal: North and Centre	-	-	chicken feces	GCF_029624415.1	Potential nonpathogenic
SAMN33901076	E. lactis F1977	Portugal: North and Centre	-	-	chicken feces	GCF_029624545.1	Potential nonpathogenic
SAMN21840343	E. lactis GX1-6	China: Hubei	-	-	feces	GCF_029600455.1	Potential nonpathogenic
SAMN21840349	E. lactis GX6-3	China: Hubei	-	-	feces	GCF_029600395.1	Potential nonpathogenic
SAMN18529541	E. lactis H53	Canada:Alberta	-	-	Beef processing facility	GCF_017942505.1	Potential nonpathogenic
SAMN21240282	E. lactis HJS001	China:Nanjing	-	-	Braised chicken	GCF_019967715.1	Potential nonpathogenic
SAMN17109561	E. lactis HPCN38	India	-	-	Common Effluent Treatment Plant (CETP) sludge	GCF_016599235.1	Potential nonpathogenic
SAMN29257247	E. lactis HPH282	Portugal	-	-	blood	GCF_026183775.1	Potential nonpathogenic
SAMN29257248	E. lactis HPH288	Portugal	-	-	blood	GCF_026183755.1	Potential nonpathogenic
SAMN21840360	E. lactis IMJ12-4	China: Hubei	-	-	Rice wine Koji	GCF_029600205.1	Potential nonpathogenic
SAMD00255146	E. lactis JCM-30200	China	-	-	Yogurt	GCF_015751065.1	Potential nonpathogenic
SAMN19988607	E. lactis JDM1	China:Shanghai	-	-	fecal sample	GCF_019203145.1	Potential nonpathogenic
SAMN32237615	E. lactis M101	South Korea	-	-	meju	GCF_028613905.1	Potential nonpathogenic
SAMN32237665	E. lactis M210h	South Korea	-	-	meju	GCF_027211905.1	Potential nonpathogenic
SAMN32237666	E. lactis M2124h	South Korea	-	-	meju	GCF_027211935.1	Potential nonpathogenic
SAMN28771987	E. lactis M212h	South Korea	-	-	Meju	GCF_023653355.1	Potential nonpathogenic
SAMN32237667	E. lactis M213h	South Korea	-	-	meju	GCF_027211985.1	Potential nonpathogenic
SAMN32237729	E. lactis M426h	South Korea	-	-	meju	GCF_027212165.1	Potential nonpathogenic
SAMN32237734	E. lactis M427	South Korea	-	-	meju	GCF_027212125.1	Potential nonpathogenic
SAMN32237784	E. lactis M5123	South Korea	-	-	meju	GCF_027212485.1	Potential nonpathogenic
SAMN32237788	E. lactis M749h	South Korea	-	-	meju	GCF_027212415.1	Potential nonpathogenic
SAMN32238061	E. lactis M750h	South Korea	-	-	meju	GCF_027212375.1	Potential nonpathogenic
SAMN32238501	E. lactis M8125	South Korea	-	-	meju	GCF_027214405.1	Potential nonpathogenic
SAMN24518866	E. lactis MG2-8	Mongolia:Tuv Province	-	-	aaruul traditional Mongolian dairy product	GCF_028888065.1	Potential nonpathogenic
SAMN08009698	E. lactis NM29-3	China: HulunBuir	-	-	Yoghurt	GCF_016767675.1	Potential nonpathogenic
SAMN08009699	E. lactis NM30-4	China: HulunBuir	-	-	Yoghurt	GCF_016767645.1	Potential nonpathogenic
SAMN08009700	E. lactis NM31-5	China: HulunBuir	-	-	Yoghurt	GCF_016767635.1	Potential nonpathogenic
SAMN31809788	E. lactis OM18-10LBA	China: Shenzhen	-	-	Homo sapiens fecal material	GCF_027691285.1	Potential nonpathogenic
SAMN28178374	E. lactis PS1156	NA	-	-	missing	GCF_024581245.1	Potential nonpathogenic
SAMN08009706	E. lactis S10-4	China: Ngawa	-	-	Fermented cattle milk	GCF_016767545.1	Potential nonpathogenic
SAMN21907283	E. lactis SCPM-O-B-8929	Russia: Moscow region	-	-	Homo sapiens genitourinary system	GCF_020405935.1	Potential nonpathogenic
SAMN21907292	E. lactis SCPM-O-B-8939	Russia: Moscow	-	-	Homo sapiens digestive system	GCF_020405675.1	Potential nonpathogenic
SAMN21907300	E. lactis SCPM-O-B-8947	Russia: Moscow	-	-	Homo sapiens digestive system	GCF_020405655.1	Potential nonpathogenic
SAMN21907301	E. lactis SCPM-O-B-8948	Russia: Moscow	-	-	Homo sapiens digestive system	GCF_020405685.1	Potential nonpathogenic
SAMN21907306	E. lactis SCPM-O-B-8953	Russia: Yaroslavl region	-	-	poultry respiratory system	GCF_020406935.1	Potential nonpathogenic

SAMN36380979	E. lactis SU-B46	Argentina: Villa Maria	-	-	milk	GCF_030490365.1	Potential nonpathogenic
SAMN08009725	E. lactis Tb32-6	China: Lhasa	-	-	Fermented yak milk	GCF_016767535.1	Potential nonpathogenic
SAMN31809328	E. lactis TM115-109	China: Shenzhen	-	-	fecal material	GCF_027679825.1	Potential nonpathogenic
SAMN31809348	E. lactis TM115-149	China: Shenzhen	-	-	fecal material	GCF_027679445.1	Potential nonpathogenic
SAMN31809447	E. lactis UN03-102	China: Shenzhen	-	-	fecal material	GCF_027676765.1	Potential nonpathogenic
SAMN31809448	E. lactis UN03-105	China: Shenzhen	-	-	fecal material	GCF_027676695.1	Potential nonpathogenic
SAMN31809450	E. lactis UN03-111	China: Shenzhen	-	-	fecal material	GCF_027676705.1	Potential nonpathogenic
SAMN31809451	E. lactis UN03-119	China: Shenzhen	-	-	fecal material	GCF_027676665.1	Potential nonpathogenic
SAMN31809454	E. lactis UN03-14	China: Shenzhen	-	-	fecal material	GCF_027676575.1	Potential nonpathogenic
SAMN31809457	E. lactis UN03-157	China: Shenzhen	-	-	fecal material	GCF_027676425.1	Potential nonpathogenic
SAMN31809459	E. lactis UN03-168	China: Shenzhen	-	-	fecal material	GCF_027676385.1	Potential nonpathogenic
SAMN31809466	E. lactis UN03-19	China: Shenzhen	-	-	fecal material	GCF_027676495.1	Potential nonpathogenic
SAMN31809487	E. lactis UN03-36	China: Shenzhen	-	-	fecal material	GCF_028826015.1	Potential nonpathogenic
SAMN31809488	E. lactis UN03-37	China: Shenzhen	-	-	fecal material	GCF_027675805.1	Potential nonpathogenic
SAMN08009727	E. lactis XJ28301	China: Ili	-	-	Yoghurt	GCF_016767575.1	Potential nonpathogenic
SAMN21840350	E. lactis XM1-2	China: Inner Mongolia	-	-	Dairy product	GCF_029600385.1	Potential nonpathogenic
SAMN21840348	E. lactis XY10-9	China: Hubei	-	-	feces	GCF_029600435.1	Potential nonpathogenic
SAMN21840344	E. lactis XY2-5	China: Hubei	-	-	feces	GCF_029600495.1	Potential nonpathogenic
SAMN21840345	E. lactis XY6-6	China: Hubei	-	-	feces	GCF_029600465.1	Potential nonpathogenic
SAMN21840346	E. lactis XY6-7	China: Hubei	-	-	feces	GCF_029600525.1	Potential nonpathogenic
SAMN08009702	E. lactis XZ35303	China: Lhasa	-	-	Fermented yak milk	GCF_016767615.1	Potential nonpathogenic
SAMN08057240	E. faecium 10-131	France: Clichy	Homo sapiens	Catheter infection	Catheter	GCF_002909305.1	Potential pathogenic
SAMN08093651	E. faecium 16-164-1	France: Chambery	Homo sapiens	Urinary tract infection	urine	GCF_002973675.1	Potential pathogenic
SAMN11980290	E. faecium 164306	Portugal: Coimbra	Homo sapiens	urinary tract infection	urine	GCF_006375995.1	Potential pathogenic
SAMN24298383	E. faecium 17-040	Reunion: Saint-Denis	Homo sapiens	Intra-abdominal infection	Peritoneal flu	GCF_021311055.1	Potential pathogenic
SAMN07725781	E. faecium 18434	Russia: St.Petersburg	Homo sapiens	nosocomial infection	blood	GCF_002562805.1	Potential pathogenic
SAMN09225716	E. faecium 19	Russia: Chelyabinsk Ural region	Homo sapiens	nosocomial infection	endotracheal aspirate	GCF_003240455.1	Potential pathogenic
SAMN32671897	E. faecium 330767	India: Puducherry	Homo sapiens	Wound infection	pus	GCF_028204055.1	Potential pathogenic
SAMN06850205	E. faecium 463-AER	USA: Pennsylvania	Homo sapiens	bacteremia	blood; aerobic bottle	GCF_002119065.1	Potential pathogenic
SAMN21850488	E. faecium 4995/20	Poland: Bydgoszcz	Homo sapiens	wound infection	hematoma	GCF_020221735.2	Potential pathogenic
SAMN12141647	E. faecium 515	USA: Houston	Homo sapiens	endocarditis	infection	GCF_006575625.1	Potential pathogenic
SAMN04397503	E. faecium 805447/07	Austria: Vienna	Homo sapiens	unknown	Pleura effusion	GCF_001563205.1	Potential pathogenic
SAMN35725530	E. faecium A2	South Korea	Homo sapiens	Bloodstream infection	blood	GCF_030316155.1	Potential pathogenic
SAMN15643624	E. faecium AML0158	Germany:Cologne	Homo sapiens	Bloodstream infection	clinical	GCF_014489995.1	Potential pathogenic
SAMN02604219	E. faecium AUS0085	Australia	Homo sapiens	missing	blood	GCF_000444405.1	Potential pathogenic
SAMN18905391	E. faecium C6	Netherlands	Homo sapiens	bloodstream infection	blood	GCF_020178155.1	Potential pathogenic
SAMN19459781	E. faecium CL10395	Brazil	Homo sapiens	Blood Infection	blood	GCF_020163015.1	Potential pathogenic
SAMN00002237	E. faecium DO	USA: University of Texas Medical School	Homo sapiens	bloodstream infection	Blood	GCF_000174395.2	Potential pathogenic
SAMN02471503	E. faecium E1162	France	Homo sapiens	bloodstream infection	bloodstream infection of a hospitalized patient	GCF_000172675.1	Potential pathogenic
SAMN04270921	E. faecium E13931	Sweden: Eskilstuna	Homo sapiens	several infections	feces	GCF_001546995.1	Potential pathogenic
SAMN09469426	E. faecium E64	Russia: Archangelsk	Homo sapiens	Urinary tract infection	urine	GCF_003284785.1	Potential pathogenic
SAMN10823987	E. faecium EF_529	USA: St. Louis MO	Homo sapiens	E. faecium infection	Infection	GCF_004151895.1	Potential pathogenic
SAMN10824006	E. faecium EF_548	Pakistan: Islamabad	Homo sapiens	E. faecium infection	Infection	GCF_004151695.1	Potential pathogenic
SAMN32302292	E. faecium EF0656	China: Zhuhai	infection	infection	urine	GCF_027286245.1	Potential pathogenic
SAMN34171214	E. faecium Efm10	Brazil: Curitiba	Homo sapiens	bacteremia	blood	GCF_030346955.1	Potential pathogenic
SAMN10252953	E. faecium HBSJRP 10	Brazil: Sao Jose do Rio Preto	Homo sapiens	Infection	Ascitic Flu	GCF_009078405.1	Potential pathogenic
SAMN11980296	E. faecium HPH9	Portugal: Porto	Homo sapiens	urinary tract infection	urine	GCF_006375865.1	Potential pathogenic
SAMN03988631	E. faecium ISMMS_VRE_12	USA: New York City	Homo sapiens	Enterococcus bacteremia	blood	GCF_001953235.1	Potential pathogenic
SAMN07326775	E. faecium K59-68	Norway	Homo sapiens	Bacteraemia	blood	GCF_002263115.1	Potential pathogenic
SAMD00202474	E. faecium KUHS13	Japan	missing	missing	missing	GCF_009938285.1	Potential pathogenic
SAMN06555614	E. faecium LIM699	Brazil: Hospital das Clinicas Sao Paulo	Homo sapiens	Bloodstream infection or infection or gastrointestinal tract colonization	blood	GCF_002027515.1	Potential pathogenic
SAMN17718671	E. faecium P69Iso2	Germany: He	Homo sapiens	bloodstream infection	blood culture	GCF_022844775.1	Potential pathogenic
SAMN10273305	E. faecium RBWH1	Australia: Brisbane	Homo sapiens	Urinary tract infection	urine	GCF_003957785.1	Potential pathogenic
SAMN02471515	E. faecium U0317	Netherlands: Utrecht	Homo sapiens	urinary tract infection	urinary tract infection of a hospitalized patient	GCF_000172915.1	Potential pathogenic
SAMN10869120	E. faecium UAMSEF_52	USA:Little Rock AR	Homo sapiens	bloodstream infection	blood	GCF_004299865.1	Potential pathogenic
SAMN27728132	E. faecium UCH5	USA	Homo sapiens	Enterococcus infection	blood	GCF_023204975.1	Potential pathogenic
SAMN10142019	E. faecium UEL170	Brazil: Parana Londrina	Homo sapiens	Urinary tract infection	urine	GCF_003697805.1	Potential pathogenic
SAMN27288985	E. faecium UMM1_1	USA: University of Minnesota Medical Center Minneapolis Minnesota	missing	missing	ventricular Assist Device	GCF_023647315.1	Potential pathogenic
SAMEA8659991	E. faecium USZ_VRE67_P60	Switzerland	Homo sapiens	Superficial wound	Superficial wound	GCF_907176815.1	Potential pathogenic
SAMN10935273	E. faecium V24	Denmark: Copenhagen	Homo sapiens	Colonisation in the gastrointestinal tract	gastrointestinal tract	GCF_004302885.1	Potential pathogenic
SAMN12818041	E. faecium V2937	Denmark:Copenhagen	Homo sapiens	Infection	Rectum	GCF_008728475.1	Potential pathogenic
SAMN11890391	E. faecium VRE1	China:Hangzhou	Homo sapiens	urinary tract infection	urine	GCF_006007925.1	Potential pathogenic
SAMN04500822	E. faecium VREr6	Malaysia	Homo sapiens	Endocarditis	blood	GCF_001969545.1	Potential pathogenic
SAMN18018081	E. lactis 102-1	China: Henan	-	-	Probiotic product	GCF_018397275.1	Potential probiotic
SAMN18018082	E. lactis 104-1	China: Henan	-	-	Probiotic product	GCF_018397285.1	Potential probiotic
SAMN18018054	E. lactis 1-1	China: Anhui	-	-	Probiotic product	GCF_018397815.1	Potential probiotic
SAMN18018080	E. lactis 97-1	China: Beijing	-	-	Probiotic product	GCF_018397325.1	Potential probiotic
SAMN18018073	E. faecium 71-1	China: Jiangsu	-	-	Probiotic product	GCF_018397465.1	Potential probiotic
SAMN20247403	E. faecium EF201	USA: Manhattan KS	-	-	Probiotic	GCF_019662495.1	Potential probiotic
SAMN20247411	E. faecium EF209	USA: Manhattan KS	-	-	Probiotic	GCF_019662305.1	Potential probiotic
SAMN20247413	E. faecium EF211	USA: Manhattan KS	-	-	Probiotic	GCF_019662325.1	Potential probiotic
SAMN20247417	E. faecium EF215	USA: Manhattan KS	-	-	Probiotic	GCF_019662185.1	Potential probiotic
SAMN20247421	E. faecium EF219	USA: Manhattan KS	-	-	Probiotic	GCF_019659155.1	Potential probiotic
SAMN20247424	E. faecium EF222	USA: Manhattan KS	-	-	Probiotic	GCF_019662165.1	Potential probiotic
SAMN03031299	E. faecium L-3	Russia: Saint-Petersburg	-	-	Probiotic preparation	GCF_000787055.1	Potential probiotic
SAMN18018063	E. lactis 26-1	China: Jiangsu	-	-	Probiotic product	GCF_018397625.1	Potential probiotic
SAMN18018055	E. lactis 3-1	China: Anhui	-	-	Probiotic product	GCF_018397835.1	Potential probiotic
SAMN18018065	E. lactis 32-1	China: Liaoning	-	-	Probiotic product	GCF_018397615.1	Potential probiotic
SAMN18018056	E. lactis 4-1	China: Beijing	-	-	Probiotic product	GCF_018397765.1	Potential probiotic
SAMN18018067	E. lactis 45-1	South Korea	-	-	Probiotic product	GCF_018397595.1	Potential probiotic
SAMN18018075	E. lactis 76-1	China: Shandong	-	-	Probiotic product	GCF_018397385.1	Potential probiotic
SAMN18018076	E. lactis 77-1	China: Shandong	-	-	Probiotic product	GCF_018397355.1	Potential probiotic
SAMN20247405	E. lactis EF203	USA: Manhattan	-	-	Probiotic	GCF_019662395.1	Potential probiotic
SAMN20247406	E. lactis EF204	USA: Manhattan	-	-	Probiotic	GCF_019662445.1	Potential probiotic
SAMN20247407	E. lactis EF205	USA: Manhattan	-	-	Probiotic	GCF_019662405.1	Potential probiotic
SAMN20247410	E. lactis EF208	USA: Manhattan	-	-	Probiotic	GCF_019662335.1	Potential probiotic
SAMN20247418	E. lactis EF216	USA: Manhattan	-	-	Probiotic	GCF_019659205.1	Potential probiotic
SAMN20247419	E. lactis EF217	USA: Manhattan	-	-	Probiotic	GCF_019662215.1	Potential probiotic

*The two strains isolated in this study

Safety assessment of *Enterococcus lactis* strains complemented with comparative genomic analysis reveals probiotic and safety characteristics of the entire species

Noha A. Ahmed*1, Rania Abdelmonem Khattab1, Yasser M. Ragab1, Mariam Hassan*1,2
1Microbiology and Immunology Department, Faculty of Pharmacy, Cairo University, Kasr Al-Aini 11562, Cairo, Egypt.
2Department of Microbiology and Immunology, Faculty of Pharmacy, Galala University, New Galala City, 43511 Suez, Egypt."

Table S2A. List of known and predicted virulence factors* with best hits for *Enterococcus* genus in *E. lactis* 10NA genome.**

Subject ID	Subject title	Query ID	Location in the genome	PGAP annotation NCBI Reference Sequence	COG_category	Percent Identidy	percent similarity	q. start	q. end	s. start	s. end	evalue	bit score	coverage
VFG045635(gb|WP_002296629)	(sgrA) cell wall anchored protein SgrA [SgrA (VF0540) - Adherence (VFC0001)] [Enterococcus faecium DO]	WP_002342601.1	**NODE_2 (13139..14086)**	WP_002296629.1 MULTISPECIES: LPXTG-anchored fibrinogen/nidogen-binding adhesin SgrA [Enterococcus]	Function unknown	92.284	94.44	1	324	**1**	315	1E-166	462	100
VFG045614(gb|WP_002286843)	(efaA) endocarditis specific antigen [EfaA (VF0354) - Adherence (VFC0001)] [Enterococcus faecium Aus0004]	WP_002329587.1	**NODE_6 (31440..32390)**	WP_002286843.1 MULTISPECIES: metal ABC transporter substrate-binding protein [Bacteria]	Inorganic ion transport and metabolis	95.57	97.47	1	316	**1**	316	0	604	100
VFG042997(gb|AAZ68040)	(srt1) Srt1 [Bee (biofilm enhancer in enterococci) (VF0746) - Adherence (VFC0001)] [Enterococcus faecalis str. E99]	WP_002291235.1	**NODE_6 (62635..63528)**	WP_002291235.1 MULTISPECIES: class C sortase [Enterococcus]	Cell wall/membrane/envelope biogenesis	50.352	71.48	3	286	**6**	286	7.9E-99	293	71
VFG043441(gb|WP_002287386)	(EFMU0317_RS16950) C40 family peptidase [Fibronectin-binding protein (VF0747) - Adherence (VFC0001)] [Enterococcus faecium U0317]	WP_142666921.1	**NODE_12 complement(22038..23606)**	WP_002310416.1 NlpC/P60 family protein [Enterococcus faecium]	Cell wall/membrane/envelope biogenesis	92.6	93.55	1	524	**1**	522	0	733	100
VFG045683(gb|WP_002294135)	(cpsB/cdsA) phosphatidate cytidylyltransferase [Capsule (VF0361) - Immune modulation (VFC0258)] [Enterococcus faecium Aus0085]	WP_002308898.1	**NODE_16 complement(31139..31939)**	WP_002294135.1 MULTISPECIES: phosphatidate cytidylyltransferase [Enterococcus]	Function unknown	99.248	100	1	266	**1**	266	0	523	100
VFG045688(gb|WP_002294134)	(cpsA/uppS) undecaprenyl diphosphate synthase [Capsule (VF0361) - Immune modulation (VFC0258)] [Enterococcus faecium Aus0004]	WP_002308897.1	**NODE_16 complement(31936..32748)**	WP_002294134.1 MULTISPECIES: isoprenyl transferase [Enterococcus]	Lipid transport and metabolism	96.296	98.15	1	270	**1**	270	0	543	100
VFG042998(gb|AAZ68041)	(srt2) Srt2 [Bee (biofilm enhancer in enterococci) (VF0746) - Adherence (VFC0001)] [Enterococcus faecalis str. E99]	WP_002329609.1	**NODE_19 complement(47729..48889)**	WP_002286364.1 MULTISPECIES: class C sortase [Enterococcus]	Cell wall/membrane/envelope biogenesis	47.059	67.49	15	333	**20**	341	2.4E-102	304	86
VFG045671(gb|WP_002294628)	(bopD) sugar-binding transcriptional regulator, LacI family [BopD (VF0362) - Biofilm (VFC0271)] [Enterococcus faecium Aus0085]	WP_002329615.1	**NODE_19 (58368..59393)**	WP_002312769.1 MULTISPECIES: LacI family transcriptional regulator [Enterococcus]	Transcription	97.626	98.81	1	337	**1**	337	0	676	99
VFG042989(gb|ACI49672)	(ACI49672) putative housekeeping sortase [PilA-type pili (PGS1, pilin gene clusters 1) (VF0744) - Adherence (VFC0001)] [Enterococcus faecium str. E1165]	WP_002338747.1	**NODE_22 (7704..8375)**	WP_002295671.1 MULTISPECIES: class A sortase [Enterococcus]	Cell wall/membrane/envelope biogenesis	100	100	1	223	**1**	223	5.8E-168	458	100
VFG042988(gb|ACI49671)	(pilA) PilA [PilA-type pili (PGS1, pilin gene clusters 1) (VF0744) - Adherence (VFC0001)] [Enterococcus faecium str. E1165]	WP_271206284.1	**NODE_22 (8428..10404)**	WP_002332682.1 SpaA isopeptide-forming pilin-related protein [Enterococcus faecium]	Cell wall/membrane/envelope biogenesis	99.392	99.54	1	658	**1**	658	0	1324	100
VFG042987(gb|ACI49670)	(ACI49670) putative pilus-dedicated sortase [PilA-type pili (PGS1, pilin gene clusters 1) (VF0744) - Adherence (VFC0001)] [Enterococcus faecium str. E1165]	WP_002316053.1	**NODE_22 (10419..11171)**	WP_002349627.1 class C sortase [Enterococcus faecium]	Cell wall/membrane/envelope biogenesis	100	100	1	250	**1**	250	0	510	100
VFG042986(gb|ACI49673)	(ACI49673) cell wall-associated LPXTG-like protein [PilA-type pili (PGS1, pilin gene clusters 1) (VF0744) - Adherence (VFC0001)] [Enterococcus faecium str. E1165]	WP_002320589.1	**NODE_22 (11187..11942)**	WP_016177596.1 pilin N-terminal domain-containing protein [Enterococcus durans]	Function unknown	100	100	1	251	**1**	251	0	508	100
VFG042985(gb|ACI49669)	(ACI49669) hypothetical hydrophobic peptide [PilA-type pili (PGS1, pilin gene clusters 1) (VF0744) - Adherence (VFC0001)] [Enterococcus faecium str. E1165]	WP_002314405.1	**NODE_22 (11955..12215)**	WP_002293489.1 MULTISPECIES: hypothetical protein [Enterococcus]	Hypothetical protein	97.674	98.84	1	86	**1**	86	3.7E-55	162	100
VFG042984(gb|ACI49668)	(ACI49668) minor pilin subunit [PilA-type pili (PGS1, pilin gene clusters 1) (VF0744) - Adherence (VFC0001)] [Enterococcus faecium str. E1165]	WP_002305225.1	**NODE_22 (12212..14302)**	WP_002346930.1 isopeptide-forming domain-containing fimbrial protein [Enterococcus faecium]	Cell wall/membrane/envelope biogenesis	99.713	100	1	696	**1**	696	0	1411	100
VFG045470(gb|AAM75247)	(cylR2) cytolysin regulator R2 [Cytolysin (VF0356) - Exotoxin (VFC0235)] [Enterococcus faecalis str. MMH594]	WP_002377051.1	**NODE_54 complement(272..475)**	WP_008787613.1 MULTISPECIES: helix-turn-helix transcriptional regulator [Firmicutes]	Transcription	46.774	69.35	3	64	**5**	66	1.61E-14	57.8	94

****Total 15 hits that pass the following criteria (similarity ≥ 60% , coverage ≥ 60%, and evalue < 1E-3)**

Table S2B. List of known and predicted virulence factors* with best hits for *Enterococcus* genus in *E. lactis* 50NA genome.***

Subject ID	Subject title	Query ID	Location in the genome	PGAP annotation NCBI Reference Sequence	COG_category	Percent Identidy	percent similarity	q. start	q. end	s. start	s. end	evalue	bit score	coverage
VFG042990(gb|ACI49664)	(ACI49664) putative pilus-dedicated sortase [PilB-type pili (PGS3) (VF0745) - Adherence (VFC0001)] [Enterococcus faecium str. E1165]	WP_002325097.1	**NODE_8 (155336..156166)**	WP_002340958.1 MULTISPECIES: class C sortase [Enterococcus]	Cell wall/membrane/envelope biogenesis	81.855	91.94	1	248	**1**	248	5.3E-143	399	89
VFG042992(gb|ACI49666)	(ACI49666) putative minor pilin subunit [PilB-type pili (PGS3) (VF0745) - Adherence (VFC0001)] [Enterococcus faecium str. E1165]	WP_086291220.1	**NODE_8 (151949..153370)**	WP_002286054.1 MULTISPECIES: SpaH/EbpB family LPXTG-anchored major pilin [Enterococcus]	Function unknown	98.943	99.15	1	473	**1**	473	0	957	100
VFG042993(gb|ACI49667)	(ACI49667) putative pilus tip protein [PilB-type pili (PGS3) (VF0745) - Adherence (VFC0001)] [Enterococcus faecium str. E1165]	WP_086291219.1	**NODE_8 (148494..151946)**	WP_002327683.1 SpaA isopeptide-forming pilin-related protein [Enterococcus faecium]	Cell wall/membrane/envelope biogenesis	86.78	90.75	77	1129	**95**	1150	0	1857	93
VFG042989(gb|ACI49672)	(ACI49672) putative housekeeping sortase [PilA-type pili (PGS1, pilin gene clusters 1) (VF0744) - Adherence (VFC0001)] [Enterococcus faecium str. E1165]	WP_002311526.1	**NODE_20 (6082..6753)**	WP_002295671.1 MULTISPECIES: class A sortase [Enterococcus]	Cell wall/membrane/envelope biogenesis	98.655	99.1	1	223	**1**	223	5.9E-165	450	100
VFG045671(gb|WP_002294628)	(bopD) sugar-binding transcriptional regulator, LacI family [BopD (VF0362) - Biofilm (VFC0271)] [Enterococcus faecium Aus0085]	WP_002312769.1	**NODE_7 complement(120569..121594)**	WP_002312769.1 MULTISPECIES: LacI family transcriptional regulator [Enterococcus]	Transcription	97.626	98.52	1	337	**1**	337	0	676	99
VFG045688(gb|WP_002294134)	(cpsA/uppS) undecaprenyl diphosphate synthase [Capsule (VF0361) - Immune modulation (VFC0258)] [Enterococcus faecium Aus0004]	WP_002308897.1	**NODE_1 (105828..106640)**	WP_002294134.1 MULTISPECIES: isoprenyl transferase [Enterococcus]	Lipid transport and metabolism	96.296	98.15	1	270	**1**	270	0	543	100
VFG045683(gb|WP_002294135)	(cpsB/cdsA) phosphatidate cytidylyltransferase [Capsule (VF0361) - Immune modulation (VFC0258)] [Enterococcus faecium Aus0085]	WP_002308898.1	**NODE_1 (106637..107437)**	WP_002294135.1 MULTISPECIES: phosphatidate cytidylyltransferase [Enterococcus]	Lipid transport and metabolism	99.248	100	1	266	**1**	266	0	523	100
VFG002195(gb|WP_002399773)	(EF0818) polysaccharide lyase, family 8 [Hyaluronidase (VF0359) - Exoenzyme (VFC0251)] [Enterococcus faecalis V583]	WP_002314124.1	**NODE_4 complement(178520..180943)**	WP_002314124.1 MULTISPECIES: polysaccharide lyase 8 family protein [Enterococcus]	Cell motility	59.85	77.68	203	1004	**12**	807	0	1030	80
VFG045614(gb|WP_002286843)	(efaA) endocarditis specific antigen [EfaA (VF0354) - Adherence (VFC0001)] [Enterococcus faecium Aus0004]	WP_002316514.1	**NODE_8 (31232..32182)**	WP_002286843.1 MULTISPECIES: metal ABC transporter substrate-binding protein [Bacteria]	Inorganic ion transport and metabolism	95.253	97.47	1	316	**1**	316	0	602	100
VFG043441(gb|WP_002287386)	(EFMU0317_RS16950) C40 family peptidase [Fibronectin-binding protein (VF0747) - Adherence (VFC0001)] [Enterococcus faecium U0317]	WP_002345658.1	**NODE_5 (197845..199401)**	WP_002310416.1 NlpC/P60 family protein [Enterococcus faecium]	Cell wall/membrane/envelope biogenesis	91.651	92.98	1	524	**1**	518	0	717	100
VFG042991(gb|ACI49665)	(pilB) PilB [PilB-type pili (PGS3) (VF0745) - Adherence (VFC0001)] [Enterococcus faecium str. E1165]	WP_002325098.1	**NODE_8 (153367..155244)**	WP_002344222.1 MULTISPECIES: SpaH/EbpB family LPXTG-anchored major pilin [Enterococcus]	Cell wall/membrane/envelope biogenesis	96.96	98.56	1	625	**1**	625	0	1236	100
VFG042997(gb|AAZ68040)	(srt1) Srt1 [Bee (biofilm enhancer in enterococci) (VF0746) - Adherence (VFC0001)] [Enterococcus faecalis str. E99]	WP_002316506.1	**NODE_8 (68148..69041)**	WP_002291235.1 MULTISPECIES: class C sortase [Enterococcus]	Cell wall/membrane/envelope biogenesis	50.352	70.77	3	286	**6**	286	6.84E-98	290	71

*****Total 12 hits that pass the following criteria (similarity ≥ 60% , coverage ≥ 60%, and evalue < 1E-3)**
***Virulence Factor Database (VFDB) full protein dataset (updated on 1 september 2023)**

Safety assessment of *Enterococcus lactis* strains complemented with comparative genomic analysis reveals probiotic and safety characteristics of the entire species

Noha A. Ahmed*1, Rania Abdelmonem Khattab1, Yasser M. Ragab1, Mariam Hassan*1,2
1Microbiology and Immunology Department, Faculty of Pharmacy, Cairo University, Kasr Al-Aini 11562, Cairo, Egypt.
2Department of Microbiology and Immunology, Faculty of Pharmacy, Galala University, New Galala City, 43511 Suez, Egypt."

Table S3A. List of bacterial toxins / toxic metabolites genes* and their locations in *E. lactis* 10NA genome.

No.	Bacterial toxins/toxic metabolites	KEGG_ID	Gene Name	Query ID	Query location	PGAP annotation NCBI Reference Sequence
1	magnesium and cobalt exporter, CNNM family<br>Bacterial toxins	K03699	**tlyC**	WP_002290822.1, WP_002293437.1	**NODE_3 complement(57538..58914), NODE_9 (15968..17269)**	WP_016610209.1 hemolysin family protein [Enterococcus casseliflavus], WP_002325203.1 MULTISPECIES: hemolysin family protein [Enterococcus]
2	exfoliative toxin A/B	K11041	**eta**	WP_002291188.1	**NODE_6 complement(101315..102280)**	WP_002291188.1 MULTISPECIES: TDT family transporter [Bacteria]
3	Hemolysin III<br>Bacterial toxins	K11068	**hlyIII**	WP_002290194.1	**NODE_8 complement(60776..61429)**	WP_002347222.1 hemolysin III family protein [Enterococcus faecium]
4	Tyrosine metabolism	K22330	**Tyrosine decarboxylase [EC:4.1.1.25]**	WP_002286400.1	**NODE_7 (111111..112988)**	WP_002286400.1 MULTISPECIES: tyrosine decarboxylase [Enterococcus]

Table S3B. List of bacterial toxins / toxic metabolites genes* and their locations in *E. lactis* 50NA genome.

No.	Bacterial toxins/toxic metabolites	KEGG_ID	Gene Name	Query ID	Query location	PGAP annotation NCBI Reference Sequence
1	magnesium and cobalt exporter, CNNM family<br>Bacterial toxins	K03699	**tlyC**	WP_002309763.1, WP_002310348.1	**NODE_3 complement(81869..83170), NODE_6 complement(57380..58756)**	WP_002325203.1 MULTISPECIES: hemolysin family protein [Enterococcus], WP_016610209.1 hemolysin family protein [Enterococcus casseliflavus]
2	exfoliative toxin A/B	K11041	**eta**	WP_002312068.1	**NODE_8 complement(108271..109239)**	WP_002312068.1 MULTISPECIES: TDT family transporter [Enterococcus]
3	Hemolysin III<br>Bacterial toxins	K11068	**hlyIII**	WP_002309577.1	**NODE_11 (42525..43178)**	WP_002347222.1 hemolysin III family protein [Enterococcus faecium]
4	Tyrosine metabolism	K22330	**Tyrosine decarboxylase [EC:4.1.1.25]**	WP_271198578.1	**NODE_7 (110834..112711)**	WP_002286400.1 MULTISPECIES: tyrosine decarboxylase [Enterococcus]

***KEGG database (Release 107.1)**

Safety assessment of *Enterococcus lactis* strains complemented with comparative genomic analysis reveals probiotic and safety characteristics of the entire species

Noha A. Ahmed*1, Rania Abdelmonem Khattab1, Yasser M. Ragab1, Mariam Hassan*1,2
1Microbiology and Immunology Department, Faculty of Pharmacy, Cairo University, Kasr Al-Aini 11562, Cairo, Egypt.
2Department of Microbiology and Immunology, Faculty of Pharmacy, Galala University, New Galala City, 43511 Suez, Egypt."

Table S4A. List of antibiotic resistance genes* and their locations in *E. lactis* 10NA genome.

No.	Resistance	KEGG_ID	Gene Name	Query ID	Query location
1	Cationic antimicrobial peptide (CAMP) resistance	K03367	**dltA** D-alanine--poly(phosphoribitol) ligase subunit 1 [EC:6.1.1.13]	WP_002329011.1	**NODE_14 (32739..34253)**
2	Cationic antimicrobial peptide (CAMP) resistance, dltABCD operon	K03739	**dltB** membrane protein involved in D-alanine export	WP_002329012.1	**NODE_14 (34250..35455)**
3	Cationic antimicrobial peptide (CAMP) resistance, dltABCD operon	K03740	**dltD** D-alanine transfer protein	WP_002292357.1	**NODE_14 (35736..37001)**
4	Vancomycin resistance	K07260	**vanY** zinc D-Ala-D-Ala carboxypeptidase [EC:3.4.17.14]	WP_002329114.1	**NODE_11 complement(76998..77822)**
5	Tetracycline resistance	K08151	**tetA** MFS transporter, DHA1 family, tetracycline resistance protein	WP_002329163.1	**NODE_2 (197426..198613)**
6	Macrolide resistance	K08217	**Mef** MFS transporter, DHA3 family, macrolide efflux protein	WP_002308213.1	**NODE_6 (83166..84443)**
7	Cationic antimicrobial peptide (CAMP) resistance, dltABCD operon	K14188	**dltC** D-alanine--poly(phosphoribitol) ligase subunit 2 [EC:6.1.1.13]	WP_002292356.1	**NODE_14 (35500..35733)**
8	Cationic antimicrobial peptide (CAMP) resistance	K14205	**mprF, fmtC** phosphatidylglycerol lysyltransferase [EC:2.3.2.3]	WP_002290133.1 , WP_002293074.1	**NODE_8 complement(16688..19279), NODE_12 complement(49765..52338)**
9	Macrolide resistance	K18231	**msr, vmlR** macrolide transport system ATP-binding/permease protein	WP_002329826.1	**NODE_12 complement(79989..81467)**
10	Aminoglycoside resistance	K18815	**aac6-I** aminoglycoside 6'-N-acetyltransferase I [EC:2.3.1.82]	WP_002293569.1	**NODE_13 complement(69774..70322)**
11	Macrolide resistance	K19350	**Lsa** lincosamide and streptogramin A transport system ATP-binding/permease protein	WP_002329522.1	**NODE_17 complement(46669..48171)**

Table S4B. List of antibiotic resistance genes* and their locations in *E. lactis* 50NA genome.

No.	Resistance	KEGG_ID	Gene Name	Query ID	Query location
1	Cationic antimicrobial peptide (CAMP) resistance	K03367	**dltA** D-alanine--poly(phosphoribitol) ligase subunit 1 [EC:6.1.1.13]	WP_086307461.1	**NODE_6 complement(191377..192891)**
2	Cationic antimicrobial peptide (CAMP) resistance, dlt ABCD operon	K03739	**dltB** membrane protein involved in D-alanine export	WP_002314225.1	**NODE_6 complement(190175..191380)**
3	Cationic antimicrobial peptide (CAMP) resistance, dlt ABCD operon	K03740	**dltD** D-alanine transfer protein	WP_002316928.1	**NODE_6 complement(188629..189894)**
4	Vancomycin resistance	K07260	**vanY** zinc D-Ala-D-Ala carboxypeptidase [EC:3.4.17.14]	WP_002344160.1	**NODE_15 complement(9630..10454)**
5	Tetracycline resistance	K08151	**tetA** MFS transporter, DHA1 family, tetracycline resistance protein	WP_271197181.1	**NODE_1 (481609..482796)**
6	Macrolide resistance	K08217	**Mef** MFS transporter, DHA3 family, macrolide efflux protein	WP_002313361.1	**NODE_8 (88426..89703)**
7	Cationic antimicrobial peptide (CAMP) resistance, dlt ABCD operon	K14188	**dltC** D-alanine--poly(phosphoribitol) ligase subunit 2 [EC:6.1.1.13]	WP_002310246.1	**NODE_6 complement(189897..190130)**
8	Cationic antimicrobial peptide (CAMP) resistance	K14205	**mprF, fmtC** phosphatidylglycerol lysyltransferase [EC:2.3.2.3]	WP_002310438.1, WP_002313892.1	**NODE_5 (168664..171237), NODE_9 (35430..38021)**
9	Multidrug resistance, efflux pump AbcA	K18104	**abcA, bmrA** ATP-binding cassette, subfamily B, bacterial AbcA/BmrA [EC:7.6.2.2]	WP_010725922.1	**NODE_26 complement(3206..4909)**
10	Aminoglycoside resistance	K18815	**aac6-I** aminoglycoside 6'-N-acetyltransferase I [EC:2.3.1.82]	WP_002293989.1	**NODE_4 complement(69928..70476)**
11	Macrolide resistance	K19350	**Lsa** lincosamide and streptogramin A transport system ATP-binding/permease protein	WP_002296175.1	**NODE_2 complement(287262..288764)**

***KEGG database (Release 107.1)**

Safety assessment of *Enterococcus lactis* strains complemented with comparative genomic analysis reveals probiotic and safety characteristics of the entire species

Noha A. Ahmed*1, Rania Abdelmonem Khattab1, Yasser M. Ragab1, Mariam Hassan*1,2
1Microbiology and Immunology Department, Faculty of Pharmacy, Cairo University, Kasr Al-Aini 11562, Cairo, Egypt.
2Department of Microbiology and Immunology, Faculty of Pharmacy, Galala University, New Galala City, 43511 Suez, Egypt."

Table S5A. List of Plasmids* and their metadata in *E. lactis* 10NA genome.

Plasmid	Number of Contigs	Size	rep_type(s)	rep_type_accession(s)	relaxase_type(s)	relaxase_type_accession(s)	mpf_type	mpf_type_accession(s)	orit_type(s)	orit_accession(s)	predicted_mobility	mash_nearest_neighbor	mash_neighbor_distance	mash_neighbor_identification
AD907	**1**	####	**rep_cluster_893**	002374__CP004064_00076	MOBP	NC_021995_00038	MPF_T	NC_020208_00096	-	-	conjugative	CP006031	0.0246831	Enterococcus faecium T110
AD269	**4**	####	**-**	-	-	-	-	-	-	-	non-mobilizable	CP034947	0.0494626	Enterococcus faecium
AC727	**11**	####	**-**	-	-	-	-	-	-	-	non-mobilizable	CP043485	0.0494626	Enterococcus faecium
AD710	**1**	9768	**-**	-	-	-	-	-	-	-	non-mobilizable	CP016742	0.054149	Lactococcus lactis subsp. cremoris
AB756	**3**	####	**rep_cluster_185**	001274__DQ198088_00024	-	-	-	-	-	-	non-mobilizable	NC_016009	0.0349268	Enterococcus faecium

Table S5B. List of Plasmids* and their metadata in *E. lactis* 50NA genome.

Plasmid	Number of Contigs	Size	rep_type(s)	rep_type_accession(s)	relaxase_type(s)	relaxase_type_accession(s)	mpf_type	mpf_type_accession(s)	orit_type(s)	orit_accession(s)	predicted_mobility	mash_nearest_neighbor	mash_neighbor_distance	mash_neighbor_identification
AD908	**10**	####	**Inc18,rep_cluster_893**	AB290882,CP003586	-	-	-	-	-	-	non-mobilizable	CP034948	0.0480354	Enterococcus faecium
AA891	**2**	8314	**rep_cluster_1249**	000557__AB158402	-	-	-	-	-	-	non-mobilizable	CP032309	0.0276889	Enterococcus faecium

***MOB-Recon: Type contigs and extract plasmid sequences (Galaxy Version 3.0.3+galaxy0)**

Safety assessment of *Enterococcus lactis* strains complemented with comparative genomic analysis reveals probiotic and safety characteristics of the entire species

Noha A. Ahmed*1, Rania Abdelmonem Khattab1, Yasser M. Ragab1, Mariam Hassan*1,2

1Microbiology and Immunology Department, Faculty of Pharmacy, Cairo University, Kasr Al-Aini 11562, Cairo, Egypt.

2Department of Microbiology and Immunology, Faculty of Pharmacy, Galala University, New Galala City, 43511 Suez, Egypt."

Table S6A. List of prophages* and their locations in *E. lactis* 10NA genome genome.

Region	Prophage size	Completeness of prophage	Total number of genes	Location
1	19.1Kb	intact	24	**NODE_1:920-20056**
2	22Kb	incomplete	25	**NODE_1:18628-40713**
3	35.1Kb	intact	60	**NODE_14:43600-78730**
4	4.1Kb	incomplete	7	**NODE_27:614-4724**
5	8.9Kb	incomplete	10	**NODE_36:1371-10324**

Table S6B. List of prophages* and their locations in *E. lactis* 50NA genome genome.

Region	Prophage size	Completeness of prophage	Total number of genes	Location
1	39Kb	intact	64	**NODE_1:251-39273**
2	9.3Kb	incomplete	11	**NODE_1:53756-63146**
3	10.9Kb	incomplete	12	**NODE_8:118899-129850**
4	37.6Kb	incomplete	25	**NODE_18:582-14892**

***PHASTER web server (https://phaster.ca/)**

Safety assessment of *Enterococcus lactis* strains complemented with comparative genomic analysis reveals probiotic and safety characteristics of the entire species

Noha A. Ahmed*1, Rania Abdelmonem Khattab1, Yasser M. Ragab1, Mariam Hassan*1,2

1Microbiology and Immunology Department, Faculty of Pharmacy, Cairo University, Kasr Al-Aini 11562, Cairo, Egypt.

2Department of Microbiology and Immunology, Faculty of Pharmacy, Galala University, New Galala City, 43511 Suez, Egypt."

Table S7A. List of non reduntant IS elements* with best hits in *E. lactis* 10NA genome.**

IS element	IS Family	Accession number	Query ID	Percent Identidy	Length	q. start	q. end	s. start	s. end	evalue	bit score	coverage
IS1485	IS3	AF029727	NZ_JANQBF010000066.1	99.927	1365	1	1366	**1366**	1	0	2460	100
IS1542	IS256	AF114715	NZ_JANQBF010000053.1	99.924	1323	1	1324	**560**	1883	0	2384	100
ISLgar5	IS256	AKFO01000017.1	NZ_JANQBF010000026.1	98.205	1313	1	1336	**25608**	24272	0	2300	100
ISLmo19	IS6	CP068981	NZ_JANQBF010000080.1	91.935	570	35	654	**619**	1	0	890	77
ISLmo21	IS3	CP068981	NZ_JANQBF010000032.1	99.84	1245	1	1247	**483**	1729	0	2241	100
ISEfa10	IS3	FJ795373	NZ_JANQBF010000037.1	98.847	1543	1	1561	**631**	2191	0	2735	100
ISEnfa4	IS256	JQ911741	NZ_JANQBF010000053.1	91.472	1212	1	1324	**560**	1883	0	1874	100
IS1252	IS30	L38972	NZ_JANQBF010000036.1	99.718	1062	1	1065	**6918**	7981	0	1904	100
ISS1W	IS6	M37396	NZ_JANQBF010000080.1	98.447	634	10	653	**644**	1	0	1117	80
ISEf1	IS256	NC_004668	NZ_JANQBF010000036.1	99.701	1334	1	1338	**6863**	5526	0	2396	100
ISEfa13	IS256	NZ_AAAK03000022	NZ_JANQBF010000068.1	92.175	1119	102	1315	**1214**	1	0	1762	92
IS1297	IS6	U59101	NZ_JANQBF010000031.1	99.01	800	1	808	**14164**	13357	0	1422	100
IS1216	IS6	X81654	NZ_JANQBF010000080.1	98.602	635	10	653	**644**	1	0	1122	80
IS1062	IS30	X96976	NZ_JANQBF010000059.1	99.812	1060	1	1062	**1189**	128	0	1907	100

Total 14 hits that pass the following criteria (Identidy ≥ 90% , coverage ≥ 60%, and evalue < 1E-5)

Table S7B. List of non reduntant IS elements* with best hits in *E. lactis* 50NA genome.***

IS element	IS Family	Accession number	Query ID	Percent Identidy	Length	q. start	q. end	s. start	s. end	evalue	bit score	coverage
ISEfm1	IS982	AF138282	NZ_JANQBE010000046.1	99.687	955	13	970	**1**	957	0	1711	92
ISLpl1	IS30	AF459445	NZ_JANQBE010000022.1	92.638	906	74	1043	**4424**	5401	0	1443	93
ISLgar5	IS256	AKFO01000017.1	NZ_JANQBE010000019.1	92.1	886	375	1336	**1**	962	0	1393	72
ISEfa5	ISL3	AY495588	NZ_JANQBE010000019.1	94.716	1434	1	1514	**8227**	6715	0	2367	100
ISEfm2	IS256	AY887085	NZ_JANQBE010000047.1	99.535	856	375	1234	**859**	1	0	1530	65
ISLmo19	IS6	CP068981	NZ_JANQBE010000049.1	93.723	642	81	765	**1**	685	0	1042	85
ISEfa11	ISL3	FJ866609	NZ_JANQBE010000019.1	98.811	1496	1	1514	**8227**	6715	0	2646	100
ISLmo14	IS6	KY613742.1	NZ_JANQBE010000001.1	99.261	806	1	812	**397807**	398618	0	1438	100
IS6770	IS30	L28754	NZ_JANQBE010000035.1	98.967	1054	1	1065	**1525**	461	0	1872	100
ISS1W	IS6	M37396	NZ_JANQBE010000049.1	95.28	646	80	757	**1**	678	0	1079	84
IS1297	IS6	U59101	NZ_JANQBE010000023.1	98.871	613	189	808	**129**	748	0	1087	77
IS1216	IS6	X81654	NZ_JANQBE010000049.1	98.54	675	80	764	**1**	685	0	1191	85

Total 12 hits that pass the following criteria (Identidy ≥ 90% , coverage≥ 60%, and evalue < 1E-5)

***ISfinder (https://www-is.biotoul.fr/index.php) (Last Database Update : 2023-07-25)**
